# Trajectories of Burden or Benefits of Caregiving Among Informal Caregivers of Older Adults: A Systematic Review

**DOI:** 10.1093/geroni/igaf014

**Published:** 2025-02-09

**Authors:** Yongjing Ping, Jeremy Lim-Soh, Truls Østbye, Shamirah D/O A’Azman, Yong Ting, Rahul Malhotra

**Affiliations:** Health Services and Systems Research, Duke-NUS Medical School, National University of Singapore, Singapore; Centre for Ageing Research and Education (CARE), Duke-NUS Medical School, National University of Singapore, Singapore; Health Services and Systems Research, Duke-NUS Medical School, National University of Singapore, Singapore; Department of Family Medicine and Community Health, Duke University, Durham, North Carolina, USA; Duke-NUS Medical School, National University of Singapore, Singapore; Duke-NUS Medical School, National University of Singapore, Singapore; Health Services and Systems Research, Duke-NUS Medical School, National University of Singapore, Singapore; Centre for Ageing Research and Education (CARE), Duke-NUS Medical School, National University of Singapore, Singapore

**Keywords:** Caregiving experiences, Informal caregivers, Long-term care

## Abstract

**Background and Objectives:**

Informal caregiving for older adults can be both burdensome and beneficial. Given that the informal caregiving situation may evolve over time, and care needs of older adults can result from diverse health conditions, it is valuable to understand the trajectories of burden or benefits of caregiving and how these trajectories vary across health conditions common among older care-recipients. This review is the first to summarize the literature on trajectories of burden or benefits of caregiving, including caregiver and care-recipient characteristics associated with the trajectories.

**Research Design and Methods:**

We reviewed longitudinal observational quantitative studies, from 5 bibliographic databases, that assessed burden or benefits of caregiving at 3 or more time points among informal caregivers of older adults (60 years or above).

**Results:**

The narrative synthesis included 41 studies, with only 7 (17%) considering trajectories of benefits. A stable average trajectory of burden or benefits of caregiving was the most common pattern over time across various care-recipient health conditions. However, an increasing burden over time was primarily observed among caregivers of persons with dementia, while a decreasing burden was noted among caregivers of persons discharged from the hospital after an acute health event. Only 6 (10%) studies, which reported heterogeneity in the progression of burden or benefits separately or jointly, identified distinctive trajectories within the same set of caregivers. Risk factors consistently identified to be associated with trajectories indicating persistently higher burden or persistently lower benefits included more care-recipient functional limitations and behavioral problems, being a non-spousal caregiver, being a solo caregiver, and perceiving less self-efficacy or competence.

**Discussion and Implications:**

Future studies should focus on the trajectories of benefits of caregiving, untangle heterogeneity in trajectories of burden or benefits of caregiving, and consider both burden and benefits concurrently to identify factors that both enhance benefits and alleviate burden over time.

**Translational Significance:** This review is the first on trajectories of burden or benefits of caregiving among informal caregivers of older adults across various health conditions. It finds the most common trajectory of burden or benefits among included studies to be stable over time. Only a few studies look at the benefits or untangle the heterogeneity in the trajectories, separately or jointly, of burden or benefits. Key care-recipient (e.g., functional limitations) and caregiver (e.g., solo caregiving) characteristics associated with persistently higher burden or persistently lower benefits are identified. This review also suggests new research directions, contributing to reducing burden or increasing benefits.

## Background and Objectives

Informal caregivers—family members or friends who provide unpaid assistance with basic or instrumental activities of daily living (ADLs/IADLs) for individuals with chronic diseases or disabilities (Roth et al., 2015)—play an essential role in long-term care (United Nations Economic Commission for Europe, 2019). With the increase in life expectancy and the gap between life and health expectancy at older ages (World Health Organization, 2020), informal care needs of the older population, as well as care intensity, will continue to grow.

Systematic reviews and meta-analyses show that caregivers have greater depressive symptoms and lower quality of life than non-caregivers (Janson et al., 2022; Magaña et al., 2020). Based on conceptual frameworks commonly used for understanding the caregiving experience, such as the stress process model or modified stress appraisal model (Pearlin et al., 1990; Verbakel et al., 2016), caregivers’ increased vulnerability may be due to the burden of caregiving. Defined as the level of multifaceted strain perceived by caregivers from caring for other persons, such as fatigue, emotional distress, schedule disruption, and social isolation (Liu et al., 2020), burden of caregiving is usually assessed using self-reported measures, such as the Zarit Burden Interview (ZBI) or negative domains of the Caregiver Reaction Assessment (Scholten et al., 2021). Care-recipients of caregivers perceiving more burden of caregiving are also likely to have poorer outcomes, such as early institutionalization (Luppa et al., 2008).

At the same time, informal caregiving can be also beneficial and rewarding (Carbonneau et al., 2010). Benefits of caregiving have been defined as the extent of psychological and social rewards perceived by caregivers due to caregiving, including the development of a close relationship with the care-recipient and satisfaction or meaning from the daily caregiving routine (Carbonneau et al., 2010). It is also usually assessed through self-reported measures, such as the Positive Aspects of Caregiving (PAC) Scale (Scholten et al., 2021). Caregivers perceiving more benefits of caregiving have better quality of life and less depressive symptoms (Quinn & Toms, 2018), and a lower desire for care-recipient institutionalization (Fields et al., 2019). A moderating role of benefits of caregiving on the association between burden of caregiving and caregiver depressive symptoms has also been reported (Wong et al., 2019), suggesting that enhancing benefits of caregiving may help alleviate the detrimental effect of burden on caregiver health outcomes.

Given the importance of burden as well as benefits of caregiving for both caregiver and care-recipient outcomes, it is of interest to summarize the literature on burden or benefits of caregiving. In doing so, it is important to take a longitudinal perspective. Burden or benefits of caregiving may evolve over time since caregivers often provide care for an extended period (The National Alliance for Caregiving, 2020) and care-recipient health conditions may progress over time (Koca et al., 2017). For instance, nearly one in two caregivers of persons with dementia are in this role for 1–3 years (Alzheimer’s Association, 2024), and their caregiving intensity may increase with increase in care-recipients’ behavior impairments over time (Koca et al., 2017). Furthermore, it is preferable to focus only on longitudinal studies with three or more time points that allow for the inclusion of curvilinear trajectories in contrast to studies with only two time points which only allow delineation of linear change. Caregivers may adapt the intensity of caregiving over time, such as using more coping strategies (DiBartolo & Soeken, 2003). Such adaptation in caregiving may reflect in trajectories of burden or benefits as a curvilinear decline or increase, respectively. A comprehensive understanding of the trajectories of burden or benefits of caregiving among caregivers of older adults will help identify vulnerable caregivers with less favorable trajectories indicating persistently higher or increasing burden, or persistently lower or increasing benefits, and factors associated with such trajectories.

A relatively recent systematic review summarized studies on trajectories of burden of caregiving among informal caregivers of persons with dementia (Van Den Kieboom et al., 2020), without considering benefits of caregiving. The review shows that increasing burden over time is common among such caregivers, which may be explained by increasing care demands with dementia progression. Interventions at early stages of caregiving, regular screening for burden of caregiving, and increasing the extent of formal care (e.g., caregiver training or respite care) over time may thus be pertinent for caregivers of persons with dementia. However, caregiving also occurs for other care-recipient health conditions, such as stroke (Mackenzie & Greenwood, 2012), cancer (Junkins et al., 2020), ADL/IADL limitations (Sung, Goh, et al., 2022), or after certain acute events like surgeries (Stabile et al., 2022). Extending the examination of the progression of burden of caregiving to a broader range of care-recipients, beyond those with dementia, will provide evidence on whether trajectories indicating an increasing burden are common across care-recipient health conditions. If such trajectories are the norm, then strategies to support caregivers of persons with dementia (i.e., early, regular, and increasing support) should be considered for those of care-recipients with other health conditions. However, if other trajectories, such as those indicating stability or decline over time are common, then the timing and scaling up or down of interventions for caregivers of older adults should vary accordingly over time. Additionally, existing reviews, focusing on identifying factors associated with burden or benefits of caregiving, include a substantial number of cross-sectional studies (Junkins et al., 2020; Mackenzie & Greenwood, 2012). Although cross-sectional evidence is informative, a focus on longitudinal studies can identify factors that can be considered as predictors rather than correlates (Conde-Sala et al., 2014a).

Concurrent consideration of studies assessing trajectories of burden or benefits of caregiving in the same review will also help identify studies that investigate co-progression of burden and benefits. A recent review, though based only on cross-sectional studies, showed that burden and benefits of caregiving have a weak to moderate negative or inverse relationship, indicating higher burden is associated with lower benefits among informal caregivers of older adults (Prakobchai, 2021). However, other recent cross-sectional studies do show a paradoxical positive relationship between burden and benefits among a proportion of informal caregivers, such that they experience high levels of burden and benefits simultaneously (Sung, Goh, et al., 2022; Sung, Hashim, et al., 2022). The inclusion of studies examining co-progression of burden and benefits of caregiving will help reveal distinctive joint trajectories of burden and benefits, including less favorable joint trajectories (e.g., increasing burden with decreasing benefits) and paradoxical joint trajectories (e.g., increasing burden but with increasing benefits) and their predictors.

This review aims to summarize the evidence on trajectories of burden or benefits of caregiving and their associated factors (caregiver or care-recipient characteristics) among informal caregivers of older adult care-recipients, without restricting the range of care-recipient health conditions. It also highlights research limitations within this field that merit further investigation.

## Research Design and Methods

This systematic review complies with the Preferred Reporting Items for Systematic Reviews and Meta-Analysis (PRISMA) statement (Moher et al., 2009). It was registered on the International Platform of Registered Systematic Review and Meta-analysis Protocols (INPLASY; Registration number: INPLASY2023100093). Ethical approval was not required as it only included published data.

### Eligibility Criteria

#### Study type

Longitudinal observational studies with quantitative data collected from the same participants at three or more time points were included. Review and meta-analyses, qualitative studies, cross-sectional studies, case–control studies, longitudinal studies with only two time points, longitudinal studies using survival analysis, intervention studies, and case studies were excluded.

#### Measures of interest: burden or benefits of caregiving

Studies that used one or more measures to quantify the burden or benefits of caregiving, such that the measures enabled the attribution of the assessed burden or benefits to informal caregiving (Malhotra et al., 2012; Tarlow et al., 2004), were included. For example, studies using the PAC Scale (Tarlow et al., 2004), in which all items have the leading phrase “Providing help to care recipient…,” indicating that the perceived benefits could be tied to assisting the care-recipient, were included. However, studies which only presented data from a measure not contextualized to informal caregiving, such as the Center for Epidemiological Studies-Depression (CES-D) scale for depressive symptoms, were not included as the constituent items could not be attributed directly to informal caregiving.

#### Informal caregivers and care-recipients

Studies whose participants comprised one or more types of informal caregivers who provided unpaid care for a person with chronic disease or disability (Roth et al., 2015), with care-recipients being noninstitutionalized older adults, aged 60 years or above, were included. This age threshold was based on the UN definition of older persons (United Nations, 2019). No additional restrictions were placed on the health condition(s) of the care-recipients. Studies pertaining to institutionalized care-recipients (at baseline or during follow-up) were excluded because existing literature has suggested that burden of caregiving is reduced after care-recipients are institutionalized (Bangerter et al., 2019). Though informal caregiving can occur in both community and institutional settings (Harper et al., 2021), conclusions about the level and shape of trajectories of burden or benefits of caregiving may be affected by inclusion of studies that have institutionalized care-recipients. However, studies where care-recipients were recruited from outpatient clinics, such as patients receiving cancer treatments, were included (Milbury et al., 2013); studies were also included if the care-recipients were recruited from acute hospitals at baseline and discharged to community settings during follow-up, such as stroke survivors (Han et al., 2017).

#### Publication types

Publications presenting empirical research, written in English, and published in peer-reviewed journals were included. Commentaries, book chapters, theses, and conference abstracts were excluded.

### Search Strategy

Five bibliographic databases—Medline (PubMed), Embase, CINAHL, PsycINFO, and The Social Science Database (ProQuest)—were searched from inception to August 22, 2024, without restriction on study period, language, and type. The search strategy, developed with support from a university librarian, comprised subject headings and keywords grouped under four concepts: informal caregivers, older care-recipients, caregiver health-related measures, and longitudinal studies. The search strategy was developed and tested in PubMed and subsequently applied to other databases (see Supplementary Table 1).

### Screening

Identified studies were de-duplicated via Endnote 20 and Rayyan (Ouzzani et al., 2016). Retained records were then screened in the Rayyan platform. Five authors, separated into four pairs (J. Lim-Soh/Y. Ping, Y. Ting/Y. Ping, S. D/O A’Azman/R. Malhotra, and Y. Ping/R. Malhotra), conducted the title/abstract screening, using the eligibility criteria listed above. To ensure consensus on the eligibility criteria among reviewers, a pilot screening of 100 randomly selected records was conducted. Subsequently, the remaining records were split between the four pairs. Each member of a pair screened the records allotted to the pair independently and was blinded until all allotted records were screened. Decision conflicts were resolved through group meetings. Y. Ping screened the full texts of all retained records using the same eligibility criteria. The reference lists of identified systematic reviews and included studies were also screened for relevant records.

The eligibility criterion for the constructs of interest (i.e., burden or benefits of caregiving) was not applied in the title/abstract and full-text screening. This was because detailed information on measures assessing burden or benefits of caregiving was often provided only in the methods or related citations of the full-text and confirming these details would prolong the screening process. Instead, all retained studies were checked during data extraction to confirm that they had data on burden or benefits of caregiving collected at three or more time points. Studies without such data were not included in the data synthesis. During data extraction, a few of the identified studies had mostly younger care-recipients, with a mean age below 60 years. To focus on caregivers of older care-recipients, these studies were also excluded by adopting the criterion of care-recipient mean age above 60 years from existing systematic reviews on older people (Gorman et al., 2014).

### Risk of Bias Assessment

An amended version of the Newcastle-Ottawa Scale (NOS) for cohort studies was used by Y. Ping and J. Lim-Soh to assess the quality of the included studies (see Supplementary Material Section 2; Wells et al., 2000). The scale is designed to evaluate the risk of bias across nonrandomized studies in systematic reviews and allows for modifications (Lacey et al., 2022). The modified version used in this review comprised three domains: (1) Selection (three items), evaluating representativeness of caregiver and care-recipient samples; (2) Comparability (one item), examining if delineation of trajectories of burden or benefits—either by testing the difference in mean value between time points or estimating the baseline value and slope of trajectories—controlled for covariates; and (3) Outcomes (four items), evaluating the quality of burden or benefits measures, explanation of loss to follow-up of the study cohort, and statistical adequacy for delineating trajectories of burden or benefits and identifying their associated factors. For each study, each item was scored as 0 or 1, and all items were summed to generate a total score (range: 0–8), with a higher score indicating a lower risk of bias. Studies were not excluded based on the risk of bias assessment.

### Data Extraction

Y. Ping conducted data extraction using a standardized Excel form developed via discussion with the coauthors. Basic characteristics of studies and study participants (i.e., paper title, first author’s name, publication year, recruitment location, sampling method, baseline sample size, care-recipients’ mean age, and types of caregivers) were extracted. To facilitate the interpretation of the trajectories, details about the measure(s) of burden or benefits of caregiving, including their names and score range, were also recorded.

Statistics representing trajectories of burden or benefits of caregiving were presented in three distinct ways in the studies. While a specific study could report the longitudinal data in more than one way, we extracted and presented only the most refined way, based on the statistical approach used. The least refined way was to report the mean value, with its standard deviation (*SD*), of burden or benefits of caregiving at each time point for all study participants combined. The second way was to report the baseline mean value and rate of change of the trajectory of burden or benefits of caregiving for all study participants combined; the rate of change was usually estimated by statistical models for analyzing data with repeated measures. The most refined way was to use Group-Based Trajectory Modeling (GBTM) or the Growth Mixture Modeling (GMM), which describe heterogeneity within the sample, to identify two or more groups among the study participants, each group having a distinctive trajectory of burden or benefits (or distinctive joint trajectory of burden and benefits) of caregiving. For studies using the most refined approach, the number and percentage of participants in each group were retrieved; for each group, trajectory-specific statistics, which could be either mean values and their *SD*s at each time point, or baseline value and rate of change over time, were recorded.

Data on characteristics of caregivers or care-recipients that were associated with the trajectories of burden or benefits of caregiving, consisting of their effect size and corresponding *p*-values, were extracted. Other covariates in multivariable models, if any, were also retrieved to assess the quality of the statistical analyses. We did not, however, consider caregiver or care-recipient characteristics associated with burden or benefits of caregiving separately at each time point.

Both time-invariant factors (i.e., factors measured only at baseline) and time-varying factors (i.e., variable factors measured repeatedly at each time point), that were associated with either the baseline value or the rate of change of the average trajectory of burden or benefits of caregiving in the multivariable model, were extracted. For studies using GBTM and GMM, time-invariant factors that predicted group membership, or time-varying factors that altered the trajectory of burden or benefits of caregiving of each group (Frankfurt et al., 2016), were recorded. Furthermore, for studies using GBTM and GMM, the difference in caregiver outcomes, if reported, assessed at or after the last follow-up time point, between the delineated trajectories were also recorded.

### Data Synthesis

A narrative synthesis was used. A meta-analysis was not conducted because of the substantial heterogeneity in study participant characteristics, study durations, follow-up intervals, measures of burden and/or benefits of caregiving, and statistical models utilized across studies. For studies reporting statistical significance of the change in burden or benefits of caregiving over time, their trajectory(ies) were classified into three types—“increasing,” “stable,” and “decreasing”—based on the general direction of the pattern(s). The classification of curvilinear trajectories, such as quadratic or cubic, if reported, was based on the difference in the values of the first and last time points. For instance, a quadratic trajectory of burden, which increased from baseline to an inflection point and then decreased until the end, was considered as “quadratic increase” if the extent of burden at the end was higher than that at baseline. Studies that did not report statistical significance were still presented; however, their trajectory(ies) were not further classified.

Factors associated with the trajectories were classified into four domains by adapting concepts from the modified stress appraisal model, which built upon Pearlin’s stress process model by incorporating formal and informal sources of support as moderators (Pearlin et al., 1990; Verbakel et al., 2016). The first domain was background characteristics, including sociodemographics of caregivers and care-recipients (e.g., age, gender, ethnicity, marital status, relation of caregiver with care-recipient, living conditions, education) or other factors reflecting caregiver self-efficacy (e.g., preparedness for caregiving). The second domain was primary stressors, covering cognitive impairment, ADL/IADL limitations, problematic behaviors, and neuropsychiatric symptoms of care-recipients. Sources of support, which was the third domain, included professional care use (e.g., home- or community-based services, or institutional care), and support from family members, friends, or volunteers. The last domain was caregivers’ health conditions, such as quality of life and depression.

## Results

### Study Selection

**Figure 1.**
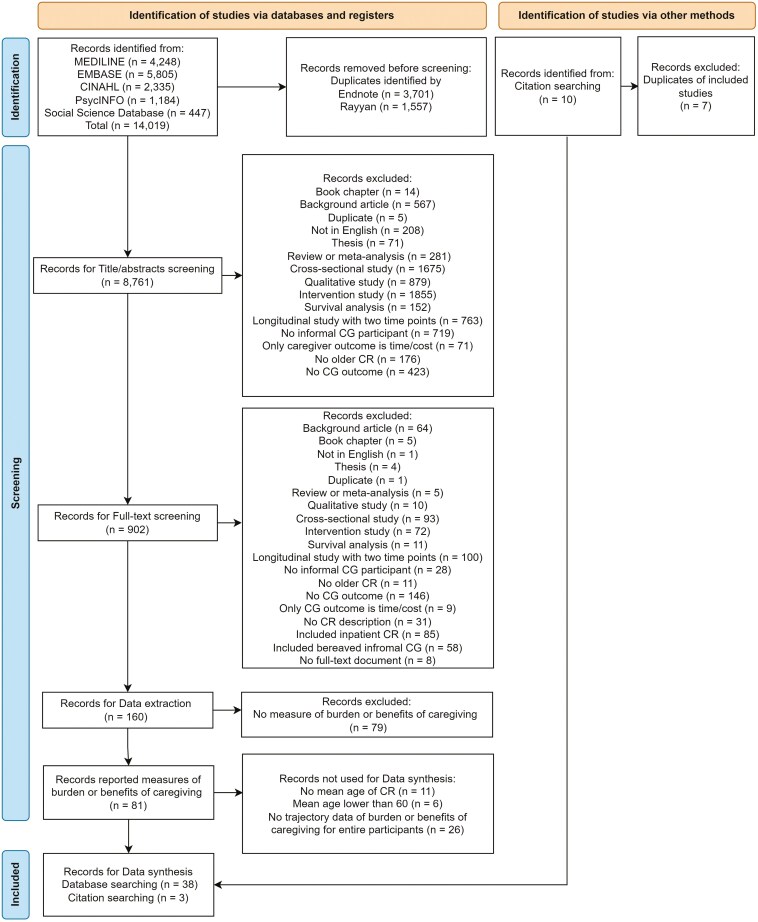
Flow diagram following the Preferred Reporting Items for Systematic Reviews and Meta-analyses (PRISMA). *Note*: CG = caregiver; CINAHL = Cumulative Index to Nursing and Allied Health Literature; CR = care-recipient; EMBASE = Excerpta Medica Database; MEDLINE = Medical Literature Analysis and Retrieval System Online.

The database search returned 14,019 records; after deduplication, 8,761 records were retained. The title/abstract and full-text screening resulted in 160 records for data extraction (Figure 1). Of these, 81 articles reporting burden or benefits of caregiving were retained. Next, 43 of the 81 articles were excluded as the care-recipients’ mean age were not reported or was less than 60 years, or no data allowing for identification of trajectories of burden or benefits of caregiving was presented, resulting in 38 studies. Searching the reference lists of identified systematic reviews and eligible studies returned three additional articles. Thus, 41 studies were included for narrative synthesis. The interrater reliability was assessed by the percent agreement between reviewers within each pair during the title/abstract screening, ranging from 91% to 94%.

### Basic Study Characteristics

Table 1 and Supplementary Table 2 present the sample characteristics and sampling strategies of the 41 included studies. Of these studies, 40 (98%) used nonprobability sampling. Only 7 of the 41 (17%) studies were conducted in Asian countries (3 in Singapore, 3 in China, and 1 in Japan). The number of caregivers at baseline ranged from 41 to 1,264. Nine of the 41 (22%) studies were exclusive to older care-recipients (i.e., all were aged 60 years or above). The 41 included studies covered a range of care-recipient health conditions, such as dementia (44%, 18 studies), cancer (17%, 7 studies), and stroke (12%, 5 studies). Spouse and adult child caregivers were the main types of informal caregivers, contributing more than 50% of the total sample in most studies (81%, 33 of 41 studies). Nineteen of the 41 studies (46%) had only three time points: in most (95%, 18 of 19 studies), the follow-up interval between consecutive time points was 3 months to 1 year. Among the other 22 studies with four or more time points, 16 (73%) studies also had a follow-up interval of 3 months to 1 year. Regarding study quality, the modified NOS scores ranged from 1 to 7 (mean [*SD*] = 4.3 [1.7], see Supplementary Table 3 and Supplementary Figure 1).

**Table 1.** Basic Characteristics of the Included Studies

Study	Country/region	Study duration	Time points (*N*)	Follow-up interval, relative to baseline (T1)	T1 CGs (*N*)	CR age (mean (*SD*))	CR diagnosis
Alspaugh et al. (1999)	United States	1.0 yr	3	T2: 3 mo, T3: 1 yr	305	77.4 (8.6)	Dementia
Bangerter et al. (2019)	United States	1.0 yr	3	T2: 6 mo, T3: 1 yr	153	82.0 (8.3)	Dementia
Bartoli et al. (2024)	Italy	1.0 yr	5	T2: 3 mo, T3: 6 mo, T4: 9 mo, T5: 12 mo	228	70.7 (NR)	Stroke
Brodaty et al. (2014)	Australia	1.0 yr	3	T2: 6 mo, T3: 1 yr	577	77.8 (7.5)	Dementia
Bryson et al. (2013)	Canada	30 d	3	T2: 7 d, T3: 30 d	102	>65 yr	After surgery
Burke et al. (2018)	Ireland	8 mo	3	T2: 4 mo, T3: 8 mo	85	63.9 (11.2)	ALS
Malhotra et al. (2024)	Singapore	2.0 yr	7	T2: 4 mo, T3: 8 mo, T4: 12 mo, T5: 16 mo, T6: 20 mo, T7: 24 mo	215	>65 yr	Dementia
Conde-Sala et al. (2014a)	Spain	3.0 yr	4	T2: 1 yr, T3: 2 yr, T4: 3 yr	330	78.0 (5.5)	Dementia
Conde-Sala et al. (2014b)	Spain	3.0 yr	4	T2: 1 yr, T3: 2 yr, T4: 3 yr	119	77.0 (6.7)	Dementia
Connors et al. (2019)	Australia	3.0 yr	6	T2: 3 mo, T3: 6 mo, T4: 1 yr, T5: 2 yr, T6: 3 yr	177	76.0 (6.8)	MCI
Connors et al. (2020)	Australia	3.0 yr	6	T2: 3 mo, T3: 6 mo, T4: 1 yr, T5: 2 yr, T6: 3 yr	720	78.0 (7.5)	Dementia
Connors et al. (2023)	Australia	3.0 yr	4	T2: 1 yr, T3: 2 yr, T4: 3 yr	164	75.8 (6.9)	MCI
Gaugler et al. (2000a)	United States	3.0 yr	4	T2: 1 yr, T3: 2 yr, T4: 3 yr	137	73.6 (8.7)	Dementia
Goldstein et al. (2006)	United Kingdom	1.0 yr (on average)	3	T2: 5–10 mo, T3: 10–22 mo	50	63.1 (10.3)	ALS
Guerriere et al. (2016)	Canada	3.6 mo (on average)	8 (on average)	2 wk until CR death	327	71.7 (12.9)	Cancer
Han et al. (2017)	China	6 mo	4	T2: 3 wk, T3: 3 mo, T4: 6 mo	164	64.0 (9.8)	Stroke
Jansen et al. (2021)	Belgium/Netherlands	3.0 yr	3	T2: 1 yr, T3: 3 yr	104	>70 yr	Cancer
Kajiwara et al. (2018)	Japan	1.0 yr	3	T2: 6 mo, T3: 1 yr	41	87.3 (7.1)	Dementia
Kellermair et al. (2021)	Australia	2.0 yr	5	T2: 6 mo, T3: 1 yr, T4: 1.5 yr, T5: 2 yr	68	71.9 (7.3)	PSP/CBS
Kuo et al. (2024)	China	2.0 yr	3	T2: 1 yr, T3: 2 yr	200	>60 yr	Dementia
Kurtz et al. (2004)	United States	1.0 yr	4	T2: 2-3 mo, T3: 5–7 mo, T4: 1 yr	491	>65 yr	Cancer
Lai (2009)	Canada	2.0 yr	3	T2: 1 yr, T3: 2 yr	111	>65 yr	ADL/IADL limitations
Lee et al. (2018)	China	6 mo	4	T2: 1 mo, T3: 3 mo, T4: 6 mo	150	60.0 (11.4)	Cancer
Li et al. (2018)	Europe	2.0 yr	5	T2: 6 mo, T3: 1 yr, T4: 1.5 yr, T5: 2 yr	1264	76.6 (7.5)	Dementia
Liu et al. (2019)	United States	1.0 yr	3	T2: 6 mo, T3: 1 yr	184	82.0 (8.3)	Dementia
Mausbach et al. (2007)	United States	5.0 yr	6	T2: 1 yr, T3: 2 yr, T4: 3 yr, T5: 4 yr, T6: 5 yr	126	77.3 (6.5)	Dementia
Milbury et al. (2013)	United States	6 mo	3	T2: 3 mo, T3: 6 mo	158	62.9 (10.1)	Cancer
Oakley et al. (2015)	United States	12 wk	4	T2: 2 wk, T3: 6 wk, T4: 12 wk	48	>65 yr	After surgery
Perales et al. (2016)	Spain	2.0 yr	3	T2: 1 yr, T3: 2 yr	221	77.8 (NR)	Dementia
Perrin et al. (2009)	United States	1.0 yr	3	T2: 6 mo, T3: 1 yr	124	66.1 (10.6)	Stroke
Pressler et al. (2013)	United States	8 mo	3	T2: 4 mo, T3: 8 mo	63	69.0 (12.6)	Heart failure
Pucciarelli et al. (2018)	Italy	1.0 yr	5	T2: 3 mo, T3: 6 mo, T4: 9 mo, T5: 1 yr	244	71.0 (12.0)	Stroke
Quinn et al. (2024)	United Kingdom	2.0 yr	3	T2: 1 yr, T3: 2 yr	1203	75.0 (7.8)	Dementia
Ransmayr et al. (2018)	Australia	2.0 yr	4	T2: 6 mo, T3: 1 yr, T4: 2 yr	585	75.7 (11.1)	Dementia
Malhotra et al. (2018)	Singapore	7.1 mo (on average)	3	T2: 11–22 wk, T3: 23–47 wk	173	71.5 (12.2)	Stroke
Saltz et al. (1999)	United States	1.0 yr	3	T2: 6 mo, T3: 1 yr	230	>65 yr	Hip fracture
Siminoff et al. (2024)	United States	22 wk	11	T2: 2 wk, T3: 4 wk, T4: 6 wk, T5: 8 wk, T6: 10 wk, T7: 12 wk, T8: 14 wk, T9: 16 wk, T10: 18 wk, T11: 20 wk, T12: 22 wk	223	61.6 (11.7)	Cancer
Snyder & Vitaliano (2020)	United States	2.0 yr	3	T2: 1 yr, T3: 2 yr	122	74.8 (8.6)	Dementia
Van Den Kieboom et al. (2023)	Netherlands	15 mo	4	T2: 1 wk, T3: 2.5 mo, T4: 15 mo	201	78.6 (8.3)	Dementia
Walker et al. (1996)	United States	3.0 yr	4	T2: 1 yr, T3: 2 yr, T4: 3 yr	130	>65 yr	ADL/IADL limitations
Wen et al. (2022)	Singapore	2.0 yr	9	T2: 3 mo, T3: 6 mo, T4: 9 mo, T5: 12 mo, T6: 15 mo, T7: 18 mo, T8: 21 mo, T9: 24 mo	346	60.0 (10.6)	Cancer

*Note*: ADL = activities of daily living; ALS = amyotrophic lateral sclerosis; CBS = corticobasal syndrome; CG = caregiver; CR = care-recipient; d = days; IADL = instrumental activities of daily living; MCI = mild cognitive impairment; mo = months; NR = not reported; PSP = progressive supranuclear palsy; wk = weeks; yr = years.

### Trajectories of Burden of Caregiving

Of the 41 studies, 37 (90%) delineated the trajectory(ies) of burden of caregiving. The most used measure, in 17 of the 37 studies (46%), was the ZBI or its modified versions. Of the 37 studies, 27 studies (73%) that reported a test of significance for either the rate of change of their trajectories or the difference in the mean values between time points are presented in Table 2 and Supplementary Table 4 (presented by care-recipient health condition). The statistics of trajectories of burden of caregiving (i.e., mean values at each time point or baseline value and rate of change over time) are presented in Supplementary Table 5. The remaining 10 studies are presented in Supplementary Table 6.

**Table 2.** Trajectories of Burden of Caregiving Among Informal Caregivers of Older Adults in the Included Studies

Study	Study duration	CR diagnosis	Measure(s) (min–max)	Statistical model for trend(s)	Trend(s)	*p*-value for trend(s)
*Studies that delineated average trajectory(ies) of overall sample*						
Bangerter et al. (2019)	1.0 yr	Dementia	RC (1–4)	LGCM	Stable	NS
			PROS (1–4)		Stable	NS
Bryson et al. (2013)	30 d	After surgery	ZBI (0–88)	GEE ^a^	Stable	NS
Conde-Sala et al. (2014b)	3.0 yr	Dementia	ZBI (22–110)	ANOVA-rm	Increase	.001
Connors et al. (2019)	3.0 yr	MCI	ZBI (0–88)	MEM ^a^	Stable	NS
Connors et al. (2020)	3.0 yr	Dementia	ZBI (0–88)	MEM ^a^	Increase	.034
Connors et al. (2023)	3.0 yr	MCI	ZBI (0–88)	MEM ^a^	Increase	<.001
	2.0 yr	Dementia			Increase	<.001
Gaugler et al. (2000a)	3.0 yr	Dementia	RC (1–4)	LGCM	Stable	NS
			PROS (1–4)		Stable	NS
Goldstein et al. (2006)	1.0 yr	ALS	CBI (0–88)	MEM ^a^	Stable	NS
Guerriere et al. (2016)	3.6 mo (on average)	Cancer	CBS-EOLC (16–64)	MLMC ^a^	Quadratic increase	<.01
Kajiwara et al. (2018)	1.0 yr	Dementia	J-ZBI_8 (0–32)	Friedman’s test	Stable	.499
Kuo et al. (2024)	2.0 yr	Dementia	RS (0–4)	GEE ^a^	Stable	NS
Kurtz et al. (2004)	1.0 yr	Cancer	CRI-is (1–4)	MEM ^a^	Decrease	<.001
			CRI-sa (1–4)		Stable	NS
Lai (2009)	2.0 yr	ADL/IADL limitations	ZBI (0–88)	Paired t-test	Increase	T2–T1: <.01 T3–T2: <.01
Li et al. (2018)	2.0 yr	Dementia	ZBI (0–88)	MEM ^a^	Increase	0.015
Liu et al. (2019)	1.0 yr	Dementia	RC (1–4)	MEM ^a^	Stable	NS
			PROS (1–4)		Stable	NS
Mausbach et al. (2007)	5.0 yr	Dementia	PROS (1–4)	MEM ^a^	Stable	NS
Milbury et al. (2013)	6 mo	Cancer	CRA-sd (1–5)	Paired *t*-test	Stable	NS
			CRA-lfs (1–5)		Increase	T2–T1: <.001 T3–T1: <.01
			CRA-hp (1–5)		Stable	NS
			CRA-fs (1–5)		Decrease	T2–T1: <.05 T3–T1: <.05
Oakley et al. (2015)	12 wk	After surgery	ZBI (0–88)	Paired *t*-test	Quadratic increase	T2–T1: .002 T4–T2: .03
			CBI (0–96)		Quadratic increase	T2–T1: <.001 T4–T2: <.001
Perales et al. (2016)	2.0 yr	Dementia	ZBI (22–110)	ANOVA	Increase	<.001
Pressler et al. (2013)	8 mo	Heart failure	OCBS-t (18–90)	ANOVA-rm	Decrease	.002
			OCBS-d (18–90)		Decrease	.002
Pucciarelli et al. (2018)	1.0 yr	Stroke	CBI (0–100)	MEM ^a^	Cubic decrease	<.001
Saltz et al. (1999)	1.0 yr	Hip fracture	CSI (0–13)	ANOVA-rm	Decrease	.013
Siminoff et al. (2024)	22 wk	Cancer	ZBI (0–88)	LGCM	Stable	NS
Van Den Kieboom et al. (2023)	15 mo	Dementia	CRA-sd (5–25)	MEM ^a^	Increase	.002
			CRA-lfs (4–20)		Increase	.038
			CRA-hp (5–25)		Increase	.001
			CRA-fs (3–15)		Stable	NS
*Studies that reported distinctive trajectories of multiple groups of overall sample*						
Conde-Sala et al. (2014a)	3.0 yr	Dementia	ZBI (22–110)	GMM	Increase (73.9% of overall sample)	.024
					Quadratic increase (13.9% of overall sample)	<.001
					Quadratic decrease (12.2% of overall sample)	<.001
Lee et al. (2018)	6 mo	Cancer	CRA (1–5)	GBTM	Stable (34.7% of overall sample)	NS
					Stable (56.0% of overall sample)	NS
					Decrease (9.3% of overall sample)	<.001
Quinn et al. (2024)	2 yr	Dementia	RSS (0–60)	GMM	Stable (8.3% of overall sample)	NS
					Increase (46.1% of overall sample)	<.05
					Increase (39.5% of overall sample)	<.05
					Increase (6.1% of overall sample)	<.05

*Notes*: ADL = activities of daily living; ALS = amyotrophic lateral sclerosis; ANOVA = analysis of variance; ANOVA-rm = analysis of variance for repeated measures; CBI = Caregiver Burden Inventory; CBS-EOLC = Caregiver Burden Scale in End-of-Life Care; C-CSI = the Chinese version of Caregiver Strain Index; CR = care-recipient; CRA = Caregiver Reaction Assessment; CRA-fs = CRA-Financial Strain subscale; CRA-hp = CRA-Health Problem subscale; CRA-lfs = CRA-Lack of Family Support subscale; CRA-sd = CRA-Schedule Disruption subscale; CRI = Caregiver Reaction Inventory; CRI-is = CRI-Impact on Schedule subscale; CRI-sa = CRI-Sense of Abandonment subscale; CSI = Caregiver Strain Index; d = days; GBTM = Group-Based Trajectory Model; GEE = generalized estimating equations; GMM = Growth Mixture Model; IADL = instrumental activities of daily living; LGCM = latent growth curve model; MCI = mild cognitive impairment; MEM = mixed-effect model; MLMC = multilevel modeling for change; mo = months; NR = not reported; NS = not significant; OCBS-d = Oberst Caregiving Burden Scale of perceived difficulty with tasks; OCBS-t = Oberst Caregiving Burden Scale of perceived time with tasks; PROS = the Pearlin’s Role Overload Scale; RC = role captivity; RS = Role Strain scale; RSS = Relative Stress Scale; SCB = screen of caregiver burden; SCQ = Sense of Competence Questionnaire; wk = weeks; yr = years; ZBI = Zarit Burden Interview; J-ZBI_8 = the Japanese version of Zarit Burden Interview.

^a^Trajectory(ies) of burden of caregiving were estimated with one or more covariates.

The 27 studies, with study durations ranging from 30 days to 5 years, included those with a single burden measure as well as those with two distinct burden measures or separate consideration of each subscale of a burden measure. Among the 27 studies, the most common care-recipient health condition was dementia (52%, 14 studies). Of the 27 studies, 24 (89%) studies delineated only a single average trajectory of burden of caregiving for all participants, while the remaining three (11%) studies identified two or more distinctive trajectories of burden within the same set of participants.

#### Studies delineating a single average trajectory for all participants

Among the 24 studies, 13 (54%) reported a stable average trajectory of burden, which was the most common pattern across various study durations and care-recipient health conditions. For instance, Bryson et al. (2013) observed a stable trajectory over 30 days among caregivers of older adults after surgery, and Goldstein et al. (2006) did so among caregivers of persons with amyotrophic lateral sclerosis over a year. Two studies among caregivers of persons with dementia also found a stable pattern over longer periods: 3 years (Gaugler et al., 2000a) or 5 years (Mausbach et al., 2007).

Ten (41%) of the 24 studies reported an increasing average trajectory of burden. Six of the studies focused on caregivers of persons with dementia. Among these six studies, five focused on the overall burden (Conde-Sala et al., 2014b; Connors et al., 2020, 2023; Li et al., 2018; Perales et al., 2016), while Van Den Kieboom et al. (2023) studied burden subdomains and found an increase in disruption in daily schedule, lack of family support, and health problems over 15 months. The other four studies pertained to caregivers of older adults who had ADL/IADL limitations (Lai, 2009), cancer (Guerriere et al., 2016; Milbury et al., 2013), or had undergone pelvic floor surgery (Oakley et al., 2015). Both Oakley et al. (2015) and Guerriere et al. (2016) observed a quadratic trend of burden; Oakley et al. (2015) observed that burden increased over the first 2 weeks and then declined until 12 weeks after surgery, and Guerriere et al. (2016) found the rate of change to increase over time until the care-recipient died.

Five (21%) of the 24 studies observed a decreasing average trajectory of burden. Two studies pertained to the period after hospital discharge of care-recipients with stroke or hip fracture (Pucciarelli et al., 2018; Saltz et al., 1999), two focused on caregivers after care-recipients received initial cancer treatment (Kurtz et al., 2004; Milbury et al., 2013), and one investigated caregivers of persons with heart failure (Pressler et al., 2013). Interestingly, Pucciarelli et al. (2018) reported a cubic trajectory with five time points over a year after discharge—a decrease over the first 3 months, followed by an increase from 3 to 9 months, a peak at 9 months, and then a decrease until 12 months. Milbury et al. (2013) and Kurtz et al. (2004) both reported a decreasing trajectory of a burden subdomain after care-recipients received initial cancer treatments. Milbury et al. (2013) observed a decrease in perceived financial strain of caregiving over 6 months, and Kurtz et al. (2004) reported that the impact on caregivers’ schedule declined over a year.

#### Studies identifying multiple, distinctive trajectories of burden of caregiving

Only 3 (11%) of the 27 studies investigated heterogeneity in the trajectories of burden within the same study cohort among caregivers of persons with dementia (Conde-Sala et al., 2014a; Quinn et al., 2024) or cancer (Lee et al., 2018). The two studies among caregivers of persons with dementia found that the burden increased for most caregivers over 2 or 3 years. Specifically, Conde-Sala et al. (2014a) identified three distinctive trajectories over a 3-year period. The most common trajectory (74% of caregivers) showed a slight increase in burden over time but was the lowest in the extent of burden. The other two trajectories differed in the pattern of change—one trajectory increased over the first 2 years and decreased slightly thereafter (14% of caregivers), while the other decreased over the first year and remained stable thereafter (12% of caregivers). Quinn et al. (2024) identified four distinctive trajectories of burden over 2 years. Three of the trajectories (92% of caregivers) depicted an increase over time; two trajectories had a similar rate of increase but varied in the extent of burden over time, being either moderate (46% of caregivers) or the lowest (40% of caregivers), while the third, with the second highest extent of burden had a faster rate of increase (6% of caregivers). The fourth trajectory depicted the highest burden, with no change, over time (8% of caregivers). Lee et al.’s (2018) study of caregivers of persons with cancer found three trajectories of burden over a 6-month period. The two most common trajectories were both stable over time but varied in the extent of burden, being either moderate (56% of caregivers) or the highest (35% of caregivers). The least common trajectory, with only 9% of caregivers, depicted the lowest burden over time but with a slightly decreasing trend. None of the three studies compared any caregiver outcome, at or after the last time point, between the delineated trajectories.

To identify the best-fitting model of GMM or GBTM, while all three studies used Bayesian information criterion (BIC) to determine the model performance, only one reported the average posterior probability to evaluate the classification of participants in the distinctive trajectories (Quinn et al., 2024). The detailed fit statistics reported in each study are presented in Supplementary Table 7.

### Trajectories of Benefits of Caregiving

Only 7 (17%) of the 41 included studies reported trajectories of benefits of caregiving. Some of the measures used included the PAC Scale, and the caregiver esteem domain of either the Caregiver Reaction Inventory or the Caregiver Reaction Assessment. Of the seven, five studies that reported the statistical significance of the rate of change of their trajectories or the difference in the mean values between time points are presented in Table 3 and Supplementary Table 8. The remaining two are presented in Supplementary Table 9. The duration of the five studies ranged from 6 months to 3 years.

**Table 3.** Trajectories of Benefits of Caregiving Among Informal Caregivers of Older Adults in the Included Studies

Study	Study duration	CR diagnosis	Measure (min–max)	Statistical model for trend(s)	Trend(s)	*p*-value for trend(s)
*Studies that delineated average trajectory(ies) of overall sample*						
Milbury et al. (2013)	6 mo	Cancer	CRA-ce (1–5)	Paired *t*-test	Stable	NS
Van Den Kieboom et al. (2023)	15 mo	Dementia	CRA-ce (7–35)	MEM	Decrease	.026
Walker et al. (1996)	3.0 yr	ADL/IADL limitations	CS (1–7)	LGCM	Stable	NS
*Study that reported distinctive trajectories of multiple groups of overall sample*						
Quinn et al. (2024)	2 yr	Dementia	PAC (9–54)	GMM	Decrease (15.2% of overall sample)	<.05
					Stable (67.5% of overall sample)	NS
					Stable (9.3% of overall sample)	NS
					Increase (3.4% of overall sample)	<.05
					Decrease (4.5% of overall sample)	<.05
Malhotra et al. (2018)	7.1 mo (on average)	Stroke	PAC (9–54)	GBTM	Decrease (41.9% of overall sample)	<.05
					Decrease (58.1% of overall sample)	<.05

*Note*: ADL = activities of daily living; CGS = Caregiver Gratification Scale; CR = care-recipient; CRA-ce = Caregiver Esteem subscale of Caregiver Reaction Assessment; CRI = Caregiver Reaction Inventory; CS = caregiving satisfaction; GBTM = Group-Based Trajectory Model; GMM = Growth Mixture Model; IADL = instrumental activities of daily living; LGCM = latent growth curve model; MEM = mixed-effect model; mo = months; NR = not reported; NS = not significant; PAC = Positive Aspects of Caregiving Scale; yr = years.

Three of the five studies reported an average trajectory of benefits of caregiving for all participants. Of these, two studies reported a stable average trajectory over either 6 months among caregivers of persons with cancer (Milbury et al., 2013) or 3 years among caregivers of persons with ADL/IADL limitations (Walker et al., 1996). The third study showed a decreasing average trajectory over 15 months among caregivers of persons with dementia (Van Den Kieboom et al., 2023).

The remaining two studies reported the heterogeneity in the trajectories of benefits among caregivers of persons with either stroke (Malhotra et al., 2018) or dementia (Quinn et al., 2024). Malhotra et al. (2018) identified two distinctive trajectories, which differed in the extent of benefits at baseline and then both slightly declined, roughly parallel with each other, over time. Quinn et al. (2024) reported five distinctive trajectories over 2 years. Two of the trajectories showed no change over time, but varied in the extent of benefits, being either moderate (68% of caregivers) or the lowest (9% of caregivers). Of the three remaining trajectories, two showed a decline in benefits over time: one trajectory depicted the highest extent of benefits with a slight decline over time (15% of caregivers), and the other showed moderate extent of benefits but with a faster rate of decline (5% of caregivers). The fifth trajectory presented moderate extent of benefits with an increase over time (3% of caregivers). Neither study compared any caregiver outcome, at or after the last time point, between the delineated trajectories. To identify the best-fitting model, both studies used BIC and average posterior probability to examine the overall model performance and classification of participants into the distinctive trajectories, respectively (details in Supplementary Table 7).

### Joint Trajectories of Burden and Benefits of Caregiving

Table 4 and Supplementary Table 10 present that 3 (7%) of the 41 included studies reported the joint trajectories of burden and benefits of caregiving among caregivers of persons with either dementia (Malhotra et al., 2024; Quinn et al., 2024) or cancer (Wen et al., 2022). In each study, the largest proportion (28%–72%) of caregivers followed a stable joint trajectory of burden and benefits of caregiving, with the extent of burden being persistently low and extent of benefits being persistently moderate over time. Furthermore, in all three studies, a group of caregivers (15%–23%) persistently had the least burden and the highest benefits over time (vs. other caregivers in the study). Besides these common joint trajectories, paradoxical trajectories were observed. In the study by Quinn et al. (2024), 15% of the caregivers had both increasing burden and benefits over time. Wen et al. (2022), delineating joint trajectories of three subdomains of burden (i.e., schedule disruption, financial strain, and lack of family support) and benefits of caregiving, found that one in four caregivers had persistently stable and the highest extent of schedule disruption and financial strain coupled with relatively high benefits over time, suggesting that they followed a joint trajectory indicating high and stable burden as well as benefits over time. To determine the best-fitting model, all these studies used the BIC score to determine the performance of their models in categorizing participants into groups with distinctive joint trajectories of burden and benefits (Supplementary Table 7).

**Table 4.** Joint Trajectories of Burden and Benefits of Caregiving Among Informal Caregivers of Older Adults in the Included Studies

Study	Study duration	CR diagnosis	Statistical model for trend(s)	Participants in each group, %	Measure of burden (min–max)	Trend(s) (*p*-value)	Measure of benefits (min–max)	Trend(s) (*p*-value)
Malhotra et al. (2024)	2 yr	Dementia	GBMTM	22.7	mCRA (1–5)	Stable (NS)	GAIN (0–40)	Stable (NS)
				28.2		Stable (NS)		Stable (NS)
				28.3		Stable (NS)		Stable (NS)
				20.8		Stable (NS)		Stable (NS)
Quinn et al. (2024)	2 yr	Dementia	GMM	72.2	RSS (0–60)	Stable (NS)	PAC (9–54)	Stable (NS)
				15.2		Stable (NS)		Decrease (<.05)
				12.5		Increase (<.05)		Increase (<.05)
Wen et al. (2022)	2 yr	Cancer	GBMTM	38.3	mCRA-ish (1–5)	Stable (NS)	mCRA-ce (1–5)	Stable (NS)
					mCRA-if (1–5)	Stable (NS)		
					mCRA-lfs (1–5)	Stable (NS)		
				20.4	mCRA-ish (1–5)	Stable (NS)	mCRA-ce (1–5)	Stable (NS)
					mCRA-if (1–5)	Stable (NS)		
					mCRA-lfs (1–5)	Stable (NS)		
				16.4	mCRA-ish (1–5)	Stable (NS)	mCRA-ce (1–5)	Stable (NS)
					mCRA-if (1–5)	Stable (NS)		
					mCRA-lfs (1–5)	Increase (<.05)		
				24.9	mCRA-ish (1–5)	Stable (NS)	mCRA-ce (1–5)	Stable (NS)
					mCRA-if (1–5)	Stable (NS)		
					mCRA-lfs (1–5)	Increase (<.05)		

*Note*: CR = care-recipient; GAIN = Gain in Alzheimer Care Instrument; GBMTM = Group-Based Multi-Trajectory Model; GMM = Growth Mixture Model; mCRA = modified Caregiver Reaction Assessment; mCRA-ce = modified Caregiver Reaction Assessment—caregiver esteem subscale; mCRA-if = modified Caregiver Reaction Assessment—impact of finance; mCRA-ish = modified Caregiver Reaction Assessment—impact on schedule and health subscale; mCRA-lfs = modified Caregiver Reaction Assessment—lack of family support subscale; NS = not statistically significant; PAC = Positive Aspects of Caregiving Scale; RSS = Relative Stress Scale; yr = years.

Only Malhotra et al. (2024) compared caregiver outcomes between joint trajectory groups, in the subset of caregivers whose care-recipient (person with severe dementia) passed away during the 2-year follow-up, at 6 months post-demise. They reported that compared to caregivers with a persistently low burden and persistently very high benefits joint trajectory, those with a persistently high burden and persistently high benefits joint trajectory had greater psychological distress and grief and lower spiritual well-being and quality of life at 6 months post-demise.

### Factors Associated With the Trajectories of Burden or Benefits of Caregiving

Supplementary Table 11 presents caregiver and care-recipient characteristics associated with the trajectories of burden or benefits of caregiving. For background characteristics, *spousal* caregivers (Conde-Sala et al., 2014a; Lee et al., 2018; Quinn et al., 2024; Siminoff et al., 2024; Van Den Kieboom et al., 2023), as well as caregivers with *more self-efficacy or competence* (Lee et al., 2018; Quinn et al., 2024) at baseline were more likely to have persistently higher burden. Caregivers with *poorer relationship quality with care-recipients* (Quinn et al., 2024; Van Den Kieboom et al., 2023) or *less self-efficacy or competence* (Lee et al., 2018; Quinn et al., 2024; Van Den Kieboom et al., 2023) at baseline were more likely to have persistently less benefits. In studies reporting joint trajectories of burden and benefits of caregiving, *spousal* caregivers (Malhotra et al., 2024; Wen et al., 2022) as well as caregivers with *less resilience or competence* (Malhotra et al., 2024; Quinn et al., 2024) at baseline were more likely to follow a joint trajectory indicating persistently higher burden and persistently lower benefits.

For primary stressors, *provision of care to care-recipients with more ADL/IADL limitations* (Bryson et al., 2013; Conde-Sala et al., 2014a; Connors et al., 2019, 2020; Kellermair et al., 2021; Kuo et al., 2024; Pucciarelli et al., 2018; Quinn et al., 2024), *more behavioral problems or neuropsychiatric symptoms* (Conde-Sala et al., 2014a; Connors et al., 2019, 2023; Kellermair et al., 2021; Quinn et al., 2024), or *poorer physical health* (Guerriere et al., 2016; Kurtz et al., 2004; Siminoff et al., 2024) were associated with persistently higher burden. A study examining the effect of care-recipient ADL/IADL function (as a time-varying factor) on altering trajectories of burden observed that *a faster decline in function* was associated with a faster increase in burden over time (Bryson et al., 2013). Another study showed that *a faster increase in care-recipient behavioral problems* was associated with a faster increase in burden over 3 years (Gaugler et al., 2000a). In the context of benefits of caregiving, Walker et al. (1996) showed that caregivers caring for care-recipients with *a faster decline in ADL/IADL function* were more likely to have a more rapid decline in benefits over time. Another study, which used GBTM to delineate two distinctive trajectories of benefits among caregivers of stroke survivors, showed that the effect of *increase in care-recipient ADL/IADL limitations* varied between the trajectories: caregivers with a trajectory of persistently higher benefits had an increase in benefits, while those with a trajectory of persistently lower benefits had a reduction in benefits (Malhotra et al., 2018). In studies reporting joint trajectories of burden and benefits, caregivers caring for a person with dementia with *more behavioral problems* at baseline were more likely to follow a joint trajectory indicating persistently higher burden and persistently lower benefits (Malhotra et al., 2024; Quinn et al., 2024).

For sources of support, *solo caregivers* (Conde-Sala et al., 2014a; Lee et al., 2018), caregivers who *used home-based care services* at the diagnosis of cancer for their care-recipients (vs. no use of such services; Connors et al., 2020), or *had no temporary replacement caregiver* (Quinn et al., 2024), or caregivers *whose care-recipients spending more days with hospice care services* (Guerriere et al., 2016) at baseline were more likely to have persistently higher burden. Caregivers who *had no temporary replacement caregiver* were also more likely to have persistently less benefits (Quinn et al., 2024). In a study reporting joint trajectories of burden and benefits, caregivers who *did not receive emotional support from family* at baseline were more likely to follow a joint trajectory indicating a persistently higher burden and relatively high benefits, while those *who used their health saving account for care-recipient treatment* at baseline were more likely to follow a joint trajectory indicating persistently more burden and persistently less benefits (Malhotra et al., 2024).

For the health conditions of caregivers, those with *poorer mental health* (Conde-Sala et al., 2014a), *more perceived pain or fatigue* (Lee et al., 2018), or *more depressive symptoms or less self-rated health* (Quinn et al., 2024) at baseline were more likely to have persistently higher burden. Caregivers *with more depressive symptoms* at baseline were more likely to have persistently less benefits (Quinn et al., 2024). One study, reporting joint trajectories of burden and benefits of caregiving (Quinn et al., 2024), showed that caregivers with *more depressive symptoms* at baseline were more likely to follow a joint trajectory indicating persistently higher burden and persistently less benefits.

## Discussion and Implications

This systematic review synthesized the literature on trajectories of burden or benefits of caregiving and their associated factors among informal caregivers of older adults, observing that a stable (i.e., no change over time) average trajectory of burden or benefits of caregiving was the most common pattern. Among studies that reported an increasing burden of caregiving, most pertained to caregivers of persons with dementia. The few studies reporting a decreasing average trend of burden of caregiving were among caregivers of persons with stroke or hip fracture, post-hospital discharge. This review also found only a few studies that revealed the heterogeneity in trajectories of burden or benefits of caregiving, separately or jointly, within the same cohort of caregivers. Overall, there were more studies reporting trajectories of burden than those reporting trajectories of benefits. Factors, showing consistent associations with persistently higher burden or persistently lower benefits, included care-recipient functional limitations and behavior impairments, being a spousal caregiver or a solo caregiver, and perceiving less self-efficacy or competence at baseline.

Three possible reasons may help explain why a stable average trajectory of burden or benefits of caregiving was the most common pattern, irrespective of care-recipient health conditions and the duration of follow-up. First, a stable average trajectory of burden or benefits for all participants may overlook the dynamic trends within participant subgroups. For example, one study included in this review, using GBTM, observed a subgroup (9% of participants) having a decreasing trend of burden, despite finding a stable average trend of burden for all participants (Lee et al., 2018). Future studies should consider using more sophisticated analytical methods, such as GBTM or GMM, to disentangle the heterogeneity in the trajectory of burden or benefits of caregiving among caregivers of older adults. Such evidence may help identify vulnerable caregivers with persistently higher or increasing burden or persistently lower or decreasing benefits over time. Second, the trajectory of overall burden or benefits of caregiving may mask the variation in trajectories of different domains contributing to the overall burden or benefits (Kajiwara et al., 2018; Milbury et al., 2013). For example, one study (Milbury et al., 2013) reported the trajectories of four domains of burden of caregiving; the sense of lacking family support increased while the perceived financial strain decreased over 6 months, and the feelings of schedule disruption and health problems due to caregiving remained stable over the same period. Further research should consider using measures with multiple domains of burden or benefits of caregiving to assess domain-specific trajectories. Third, the lack of more time points for measuring burden and benefits as well as the long intervals between time points inform that the average trajectory may also be contributing to the stable pattern by not capturing short-term fluctuations in burden or benefits of caregiving. Nonetheless, if truly reflective of the burden or benefits progression over time, the stable pattern suggests that there is no specific time when most caregivers are more vulnerable, so they may benefit from caregiver support interventions at any stage of caregiving.

This review found that studies showing an increasing trajectory of burden of caregiving primarily pertained to caregivers of persons with dementia. This finding is consistent with that of a previous review of such caregivers (Van Den Kieboom et al., 2020). A possible explanation is the increase in care needs over time resulting from progression of dementia, including the increase in behavioral or psychological symptoms of the care-recipients. This suggests that interventions at early stages of caregiving, regular check-ins and an increase in the extent of caregiver support over time may be advantageous for caregivers of persons with dementia.

We also found studies that showed decreasing average trajectory(ies) of burden of caregiving among caregivers of older care-recipients who were discharged from a hospital following treatment of an acute health condition (Pucciarelli et al., 2018; Saltz et al., 1999). This pattern may reflect the higher extent of care needs of such care-recipients in the immediate post-hospitalization period and the gradual decline in care needs as the care-recipients recuperate. This finding suggests that such caregivers may benefit from caregiver support services, such as caregiver training or respite care, in the immediate post-discharge period.

This review found only six studies demonstrating the heterogeneity in the trajectories of burden or benefits of caregiving within the set of caregivers, either separately (Conde-Sala et al., 2014a; Lee et al., 2018; Quinn et al., 2024; Malhotra et al., 2018) or jointly (Malhotra et al., 2024; Quinn et al., 2024; Wen et al., 2022). Two studies, focusing on burden among caregivers of persons with dementia (Conde-Sala et al., 2014a; Quinn et al., 2024), suggested that despite the heterogeneity, most caregivers did experience either a persistently higher extent of or an increase in burden over time. This is consistent with studies delineating a single average trajectory of burden among such caregivers, wherein an increasing average trajectory of burden was common. Of the three studies depicting distinctive joint trajectories of burden and benefits, the largest proportion of caregivers followed a joint trajectory indicating a persistently low burden and persistently moderate benefits. However, the studies did also identify caregiver with paradoxical joint trajectories, which showed either both increasing burden and benefits over time (Quinn et al., 2024), or persistently high burden and persistently high benefits (Malhotra et al., 2024; Wen et al., 2022). However, only Malhotra et al. (2024) compared caregiver outcomes, at or after the last time point, between the delineated trajectories. These findings have a few implications. First, more studies should assess heterogeneity in the trajectories of burden or benefits, separately or jointly, and their predictors, to highlight the variability in the caregiving experience. Second, future studies should identify caregiver and care-recipient characteristics that contribute to a proportion of caregivers to have less favorable trajectories of burden (persistently high or increasing over time) or benefits (persistently low or decreasing over time), or have less favorable joint trajectories (increasing or persistently high burden and decreasing or persistently high burden benefits concurrently) or paradoxical joint trajectories (increasing or persistently high burden and benefits concurrently) of burden and benefits. Third, given that joint trajectories of burden and benefits, and their predictors, have been identified only among caregivers of persons with dementia or cancer, future research studies should delineate joint trajectories among caregivers of older adults with other health conditions. Finally, studies should examine the difference in caregiver outcomes, such as health-related outcomes (e.g., quality of life or depression), between the distinctive trajectories. This will provide further validation for the identified trajectories.

According to the NOS quality assessment, 32 (78%) studies included in this review did not exclusively include older adults as care-recipients. This may be because their focus was on care-recipients with certain health condition(s), rather than their age. In addition, 29 (71%) included studies did not discuss the reasons for attrition over time and methods of handling missing data. Such information should be clearly described in further research to allow readers to judge the potential for bias in the reported findings. Nearly all (40; 98%) studies used nonprobability sampling methods, such as convenience or purposive sampling, to select participants. Nevertheless, studies that included one or more types of informal caregivers, and used a nonprobability sampling method were still given a score in the “representativeness of informal caregivers,” since the practicality of using probability sampling methods for selecting informal caregivers is limited, such as lack of sampling frames of informal caregivers.

In addition to the research gaps discussed above, we highlight others that also deserve further investigation. First, the evidence on trajectories of benefits of caregiving is relatively sparse compared to that on burden of caregiving. Given that caregivers who experience more benefits of caregiving have a better quality of life and are more willing to continue their caregiving roles (Carbonneau et al., 2010), further research need to explore how the benefits of caregiving vary over time and what factors are associated with declining or persistently lower benefits. Second, background characteristics and primary stressors, including caregiver–recipient kinship, care-recipient ADL/IADL limitations, or behavioral problems, have been extensively investigated as predictors of trajectories of burden or benefits of caregiving. However, the role of other caregiver and care-recipient characteristics with trajectories of burden or benefits, such as use of external care-related support for care-recipients (Connors et al., 2020; Guerriere et al., 2016), has received less attention. Given that informal caregivers may need more assistance from formal care services to cater for the care needs of older adults, as support from relatives may diminish due to shrinking family sizes worldwide (Esteve et al., 2024), further research should examine how utilization of supportive care services by care-recipients and informal caregivers influences the trajectory(ies) of burden or benefits of caregiving. Third, solo informal caregivers were more likely to have persistently higher burden than those with additional help (Conde-Sala et al., 2014a; Lee et al., 2018), suggesting that sharing of caregiving responsibilities among multiple caregivers may reduce burden of caregiving for primary informal caregivers. However, the number of additional caregivers, their relationship with the primary informal caregiver (e.g., spouse, children, or domestic workers), and their previous caregiving experience were not considered in the included studies. Future research should consider assessing the role of caregiving networks, including their composition and distribution of caregiving tasks, on trajectories of burden or benefits of caregiving. Fourth, despite the potential for caregiver and care-recipient characteristics, such as care-recipient ADLs/IADLs, to change over time, few studies treated them as time-varying (Bryson et al., 2013; Gaugler et al., 2000a; Malhotra et al., 2018; Walker et al., 1996). Future studies should take within-individual variations of such variables into account and investigate their role in altering trajectories of burden or benefits of caregiving. Finally, this review only found seven studies from Asian countries, despite Asia being among the most rapidly aging region worldwide (Barber & Rosenberg, 2017) and Asian countries share a cultural norm emphasizing filial piety resulting in the ubiquity of informal care for older adults in the region (Ng et al., 2016). More longitudinal studies, with three or more time points, documenting the caregiving experience in terms of burden or benefits of caregiving in Asia are needed.

There are several limitations of this review. First, we did not search the gray literature or papers written in languages than other English, which might provide additional insights. Second, the exclusion of longitudinal studies with only two time points may have led to the exclusion of some nationally representative samples of caregivers (e.g., the National Health and Aging Trends Study from the United States) and omitted factors associated with the linear change in burden or benefits of caregiving. Third, only narrative synthesis was conducted in this review. Previous meta-analysis of longitudinal studies synthesizing trajectories of health-related measures focused on the change in the measures of interest within a specific time frame, such as a period of age (e.g., change in sugar consumption among adolescents from age 13 to 30 years; Winpenny et al., 2017), a period covering a specific event (e.g., change in mental health before and after COVID-19 pandemic; Miao et al., 2023), or a period after the onset of an event (e.g., change in cognitive function among persons following the diagnosis of psychosis; Watson et al., 2022). However, with the substantial variation in the baseline situation of caregivers and care-recipients across the studies in our review, there is no fixed anchor in terms of the starting or baseline status of the care-recipients or caregivers. Thus, although a meta-analysis can offer more robust evidence, it is unsuitable in the context of our review due to the heterogeneity in the baseline situation of caregivers and care-recipients within the included studies.

In conclusion, despite the heterogeneity of studies included in this review, we found that existing longitudinal studies, with three or more time points, have largely focused on trajectories of burden of caregiving, with limited attention on trajectories of benefits of caregiving. A stable average trajectory of burden or benefits of caregiving was the most common pattern for informal caregivers. Studies showing an increasing average trajectory of burden were primarily on informal caregivers of persons with dementia. A decreasing average trajectory of burden was observed on informal caregivers of individuals discharged from the hospital due to an acute health event. Multiple characteristics of care-recipients (i.e., more ADL/IADL limitations or behavioral problems) or caregivers (i.e., being a spousal caregiver or a solo caregiver) were identified as risk factors associated with trajectories indicating a persistently higher burden or persistently lower benefits of caregiving. Only a few studies assessed the heterogeneity in the trajectories by delineating multiple and distinctive patterns of burden or benefits of caregiving over time, separately or jointly, within the same set of caregivers, and very few pertained to caregivers of older adults in Asian countries. Future longitudinal studies should focus on trajectories of benefits of caregiving and explore more risk factors associated with either persistently lower or reducing benefits over time. Further research should also explore burden and benefits of caregiving jointly over time with more inclusive samples on caregivers across care-recipient health conditions. Longitudinal studies that investigate the progression of burden or benefits of caregiving in Asian settings are also needed, given the specific cultural norms rooted in filial piety that emphasize the role of informal caregivers in providing care to older adults.

## Supplementary Material

igaf014_suppl_Supplementary_Materials

## Data Availability

For replication purposes, studies included in this review have been cited in the reference section. The main tables and supplementary materials have provided detailed results of data synthesis. A flow diagram has been demonstrated to describe the process of identifying eligible studies for this review. Other documents, such as data extraction sheet, are available upon request.
