# Supplementary material for: Trajectories of Burden or Benefits of Caregiving Among Informal Caregivers of Older Adults: A Systematic Review
Source: Innov Aging. 2025 Feb 9;9(4):igaf014. doi: 10.1093/geroni/igaf014 (PMC11986200; doi:10.1093/geroni/igaf014)
Supplement: igaf014_suppl_Supplementary_Materials [file igaf014_suppl_supplementary_materials.docx]

***Innovation in Aging* Supplementary Material: Ping et al. Trajectories of Burden or Benefits of Caregiving Among Informal Caregivers of Older Adults: A Systematic Review.**

**Section 1: Supplementary Tables and Figures**

**Supplementary Table 1: Example Search strategies used in Medline (PubMed)**

| **Concepts** | **PubMed Mesh Terms** | **Keywords (title/abstracts)** |
| --- | --- | --- |
| 1. Informal caregivers | "Caregivers"[Mesh] | "informal caregiver" OR "informal caregivers" OR "informal carer" OR " informal carers" OR "family caregiver" OR "family caregivers" OR "family carer" OR "family carers" OR "spouse caregiver" OR "spouse caregivers" OR "spouse carer" OR "spouse carers" OR "informal care" OR "unpaid care" OR "child caregiver" OR "child caregivers" OR "child carers" OR "child carer" |
| 1. Caregiver health-related measures | "Caregiver Burden" [Mesh] OR "Functional Status"[Mesh] OR "Activities of Daily Living"[Mesh] OR "Geriatric Assessment"[Mesh] OR "Quality of Life"[Mesh] OR "Healthy Lifestyle"[Mesh] OR "Health Knowledge, Attitudes, Practice"[Mesh] OR "Mental Disorders"[Mesh] OR "Models, Biopsychosocial"[Mesh] OR "Fatigue"[Mesh:NoExp] OR "Mental Fatigue"[Mesh:NoExp] OR "Cognition"[Mesh] OR "Psychosocial Functioning"[Mesh] OR "Mental Health"[Mesh] OR "Emotions"[Mesh] OR "Burnout, Psychological"[Mesh:NoExp] OR "Financial Stress"[Mesh] OR "Social Determinants of Health"[Mesh] OR "Social Factors"[Mesh] OR "Sociodemographic Factors"[Mesh] OR "Social Interaction"[Mesh] OR "Social Alienation"[Mesh] OR "Social Deprivation"[Mesh] OR "Social Environment"[Mesh] OR "Social Integration"[Mesh] OR "Social Networking"[Mesh:NoExp] OR "Online Social Networking"[Mesh] OR "Social Change"[Mesh:NoExp] OR "Marital Status"[Mesh] OR "Social Mobility"[Mesh] OR "Social Adjustment"[Mesh] OR "Social Behavior"[Mesh] OR "Social Inclusion"[Mesh] OR "Empowerment"[Mesh] OR "Social Learning"[Mesh] OR "Social Segregation"[Mesh] OR "Family Conflict"[Mesh] OR "Intergenerational Relations"[Mesh] OR "Sibling Relations"[Mesh] | satisfaction OR benefit* OR enrichment OR meaning OR spirituality OR reward OR "positive aspect*" OR "positive appraisal*" OR "positive experience*" OR "positive perception*" OR "positive impact" OR "positive outcome*" OR enjoyment* OR pleasure OR gain* OR uplift* OR strength OR esteem OR stress OR anxiety OR fatigue OR burden OR strain OR depression OR disturbance OR depressive OR challenge* OR "negative appraisal*" OR "negative aspect*" OR "negative experience*" OR "negative impact" OR "negative perception*" OR "negative outcome*" OR burn-out OR burnout OR exhaustion OR "emotional health" OR "psychological health" OR "mental health" OR "physical health" OR "well-being" OR "quality of life" OR "sleep" OR "health status" OR psychosocial OR “functional status” OR "activities of daily living" OR "activity of daily living" OR “social interaction” OR “social isolation” OR “social cohesion” OR “social determinants of health” OR “social support” OR “social integration” OR “social network” OR “marital status” OR “social health” OR “social environment” OR “caregiver outcome” OR “caregiver outcomes” |
| 1. Older adults | "Aged"[Mesh] | elderly OR "older adult*" OR "older person*" OR "older people" OR "older patient*" OR "older relative*" |
| 1. Longitudinal studies | "Longitudinal Studies"[Mesh] OR "Prospective Studies"[Mesh] OR "Follow-Up Studies"[Mesh] OR "Body-Weight Trajectory"[Mesh] | cohort OR longitudinal OR follow-up OR prospective OR  trajectory OR trajectories OR “growth curve” OR “latent curve” OR “latent class” |

*Note*. Mesh = Medical Subject Headings, Mesh: NoExp = Medical Subject Headings: No explosion. To identify relevant articles for this review, we first performed a search under each concept in PubMed. Within each concept, we combined the search results of all MeSH terms and keywords with the Boolean operator “OR”. We then combined the search results from four concepts with the Boolean operator “AND” to identify relevant articles.

**Supplementary Table 2: Additional Basic Characteristics of Included Studies**

| **Study** | **Sampling method** | **Attrition(s), relative to baseline (T1) (%)** | **Type(s) of CGs (N (%))** |
| --- | --- | --- | --- |
| Alspaugh et al. (1999) | Non-probability | T2: 12.0%; T3: 37.0% | Spouse: 79 (42.0%); adult child: 82 (44.6%); others: 27 (14.4%) |
| Bangerter et al. (2019) | Non-probability | - | Spouse: 50 (32.7%); adult child: 94 (61.4%) |
| Bartoli et al. (2024) | Non-probability | T2: 13.2%; T3: 28.5%; T4: 48.7%; T5: 52.6% | NR |
| Brodaty et al. (2014) | Non-probability | - | Spouse: 414 (71.8%); adult child: 122 (21.1%); others: 41 (7.1%) |
| Bryson et al. (2013) | Non-probability | - | Spouse: 86 (86.0%); others: 16 (14.0%) |
| Burke et al. (2018) | Non-probability | T2: 31.8%; T3: 50.6% | Spouse: 63 (74.1%); adult child: 16 (18.8%); others: 6 (7.1%) |
| C. Malhotra et al. (2024) | Non-probability | NR | Adult child: 179 (83.3%); others: 36 (16.7%) |
| Conde-Sala et al. (2014a) | Non-probability | T2: 28.8%; T3: 49.7%; T4: 64.2% | Spouse: 136 (45.8%); adult child: 161 (54.2%) |
| Conde-Sala et al. (2014b) | Non-probability | - | NR |
| Connors et al. (2019) | Non-probability | T2: 17.0%; T3: 10.7%; T4: 14.7%; T5: 27.7%; T6: 39.0% | Spouse: 136 (76.8%); adult child: 34 (19.2%); others: 7 (4.0%) |
| Connors et al. (2020) | Non-probability | T2: 11.9%; T3: 12.4%; T4: 18.2%; T5: 34.4%; T6: 48.9% | Spouse: 515 (71.5%); adult child: 150 (20.8%); others: 55 (7.7%) |
| Connors et al. (2023) | Non-probability | T2: 14.0%; T3: 25.6%; T4: 32.3% | NR |
| Gaugler et al. (2000) | Non-probability | - | Spouse: 84 (61.3%); adult child: 53 (38.7%) |
| Goldstein et al. (2006) | Non-probability | NR | Spouse: 50 (100.0%) |
| Guerriere et al. (2016) | Non-probability | NR | Spouse: 157 (48%); adult child: 125 (38.2%); others: 45 (13.8%) |
| Han et al. (2017) | Non-probability | NR | Spouse: 125 (76.2%); others: 39 (23.8%) |
| Jansen et al. (2021) | Non-probability | T2: 58.7%; T3: 68.3% | Spouse: 70 (68.0%); others: 34 (32.0%) |
| Kajiwara et al. (2018) | Non-probability | - | Spouse: 11 (26.8%); adult child: 23 (56.1%); others: 7 (17.1%) |
| Kellermair et al. (2021) | Non-probability | T2: 38.2%; T3: 48.5%; T4:63.2%; T5: 75.0% | Spouse: 42 (65.0%); others: 23 (35.0%) |
| Kuo et al. (2024) | Non-probability | T2: 27.5%; T3: 44.0% | Spouse: 34 (17.0%); adult child: 163 (81.5%); others: 3 (1.5%) |
| Kurtz et al. (2004) | Non-probability | T2: 16.7%; T3: 21.6%; T3: 28.5% | Spouse: 378 (77.0%); others: 113 (23.0%) |
| Lai (2009) | Probability | - | NR |
| Lee et al. (2018) | Non-probability | T2: 0.0%; T3: 10.0%; T4: 16.7% | Spouse: 75 (50.0%); adult child: 53 (35.3%); parents: 2 (1.3%); others: 20 (13.4%) |
| Li et al. (2018) | Non-probability | NR | Spouse: 625 (49.4%); adult child: 492 (38.9%); friends: 26 (2.1%); others: 119 (9.4%) |
| Liu et al. (2019) | Non-probability | NR | Spouse: 66 (35.9%); adult child: 109 (59.3%); sibling: 3 (1.6%); others: 6 (3.3%) |
| Mausbach et al. (2007) | Non-probability | NR | Spouse: 126 (100.0%) |
| Milbury et al. (2013) | Non-probability | T2: 1.3%; T3: 31.7% | Spouse: 158 (100.0%) |
| Oakley et al. (2015) | Non-probability | - | Spouse: 26 (54.2%); adult child: 18 (37.5%); others: 4 (8.3%) |
| Perales et al. (2016) | Non-probability | T2: 24.9%; T3: 42.5% | Spouse: 116 (52.5%); adult child: 89 (40.3%); others: 16 (7.2%) |
| Perrin et al. (2009) | Non-probability | T2: 9.7%; T3: 20.2% | NR |
| Pressler et al. (2013) | Non-probability | T2: 6.3%; T3: 15.9% | Spouse: 43 (68.0%); adult child: 13 (21.0%); others: 7 (11.0%) |
| Pucciarelli et al. (2018) | Non-probability | T2: 23.4%; T3: 35.3%; T4: 40.2%; T5: 45.5% | Spouse: 88 (36.0%); adult child: 122 (50.0%); others: 34 (15.0%) |
| Quinn et al. (2024) | Non-probability | T2: 23.8%; T3: 41.9% | Spouse: 997 (82.9%); others: 206 (17.1%) |
| Ransmayr et al. (2018) | Non-probability | T2: 29.1%; T3: 43.3%; T4: 64.4% | Spouse: 286 (49.7%); adult child: 201 (35.0%); others: 87 (15.1%) |
| R. Malhotra et al. (2018) | Non-probability | T2: 23.6%; T3: 29.8% | Spouse: 40 (23.1%); others: 133 (76.9%) |
| Saltz et al. (1999) | Non-probability | - | Spouse: 50 (22.0%); others: 180 (78.0%) |
| Siminoff et al. (2024) | Non-probability | T2: 11.2%; T3: 19.3%; T4: 28.7%; T5: 35.9%; T6: 41.3%; T7: 46.2%; T8: 50.2%; T9: 57.0%; T10: 59.2%; T11: 61.0% | Spouse: 101 (45.3%); offspring or sibling: 79 (35.5%); others: 43 (19.2%) |
| Snyder & Vitaliano (2020) | Non-probability | - | Spouse: 122 (100.0%) |
| Van Den Kieboom et al. (2023) | Non-probability | NR | Spouse: 82 (41.0%); adult child: 91 (45.0%); others: 28 (14.0%) |
| Walker et al. (1996) | Non-probability | NR | Adult child: 130 (100.0%) |
| Wen et al. (2022) | Non-probability | T2: 31.8%; T3: 33.5%; T4: 37.6%; T5: 41.0%; T6: 44.2%; T7: 48.6%; T8: 56.7%; T9: 60.7% | Spouse: 164 (47.4%); adult child: 143 (41.3%); others: 39 (11.3%) |

*Note*. CG = Caregiver, NR = Not Reported. Studies that only included participants who completed all follow-up interviews/surveys were labeled as “-”. Attrition rate of the study sample over time refers to the proportion of participants missing during the follow-up interviews compared to the baseline time point (T1).

**Supplementary Table 3: Quality Assessment rated by Newcastle-Ottawa Scale (NOS) for Cohort Study**

| **Study** | **Selection** | | | **Comparability** | **Outcome** | | | | **Overall score** |
| --- | --- | --- | --- | --- | --- | --- | --- | --- | --- |
|  | **1. Representativeness of informal caregivers** | **2. Selection of care-recipients** | **3. Ascertainment of caregiving status** | **4. Comparability of cohorts because of the design or analysis** | **5. Assessment of measures of burden and/or benefits of caregiving** | **6. Adequacy of follow up of cohorts** | **7. Assessment of longitudinal trajectories of burden or benefits of caregiving** | **8. Assessment of associations between factors associated with burden or benefits of caregiving** |  |
| Alspaugh et al. (1999) | 1 | 0 | 0 | 0 | 1 | 1 | 0 | 0 | 3 |
| Bangerter et al. (2019) | 1 | 0 | 0 | 1 | 1 | 0 | 1 | 1 | 5 |
| Bartoli et al. (2024) | 0 | 0 | 0 | 0 | 1 | 0 | 0 | 0 | 1 |
| Brodaty et al. (2014) | 1 | 0 | 0 | 0 | 1 | 1 | 0 | 0 | 3 |
| Bryson et al. (2013) | 1 | 1 | 0 | 1 | 1 | 0 | 1 | 1 | 6 |
| Burke et al. (2018) | 1 | 0 | 0 | 1 | 1 | 0 | 0 | 0 | 3 |
| C. Malhotra et al. (2024) | 0 | 1 | 1 | 1 | 1 | 0 | 1 | 1 | 6 |
| Conde-Sala et al. (2014a) | 1 | 0 | 1 | 1 | 1 | 1 | 1 | 1 | 7 |
| Conde-Sala et al. (2014b) | 1 | 0 | 1 | 0 | 1 | 1 | 0 | 0 | 4 |
| Connors et al. (2019) | 1 | 0 | 1 | 1 | 1 | 1 | 1 | 1 | 7 |
| Connors et al. (2020) | 1 | 0 | 0 | 1 | 1 | 1 | 1 | 1 | 6 |
| Connors et al. (2023) | 0 | 0 | 0 | 1 | 1 | 0 | 1 | 1 | 4 |
| Gaugler et al. (2000) | 1 | 0 | 1 | 1 | 1 | 1 | 1 | 1 | 7 |
| Goldstein et al. (2006) | 0 | 0 | 0 | 1 | 1 | 0 | 1 | 1 | 4 |
| Guerriere et al. (2016) | 1 | 0 | 0 | 1 | 1 | 0 | 1 | 1 | 5 |
| Han et al. (2017) | 1 | 0 | 0 | 1 | 1 | 0 | 0 | 0 | 3 |
| Jansen et al. (2021) | 1 | 1 | 0 | 0 | 1 | 1 | 0 | 0 | 4 |
| Kajiwara et al. (2018) | 1 | 0 | 0 | 0 | 1 | 0 | 0 | 0 | 3 |
| Kellermair et al. (2021) | 1 | 0 | 0 | 1 | 1 | 0 | 0 | 1 | 4 |
| Kuo et al. (2024) | 1 | 1 | 0 | 1 | 1 | 0 | 1 | 1 | 6 |
| Kurtz et al. (2004) | 1 | 1 | 0 | 1 | 1 | 0 | 1 | 1 | 6 |
| Lai (2009) | 0 | 1 | 1 | 0 | 1 | 0 | 0 | 0 | 3 |
| Lee et al. (2018) | 1 | 0 | 0 | 1 | 1 | 0 | 1 | 1 | 5 |
| Li et al. (2018) | 1 | 0 | 0 | 1 | 1 | 0 | 1 | 1 | 5 |
| Liu et al. (2019) | 1 | 0 | 1 | 1 | 1 | 0 | 1 | 1 | 6 |
| Mausbach et al. (2007) | 0 | 0 | 0 | 1 | 1 | 0 | 1 | 1 | 4 |
| Milbury et al. (2013) | 0 | 0 | 0 | 0 | 1 | 0 | 0 | 0 | 1 |
| Oakley et al. (2015) | 1 | 1 | 1 | 0 | 1 | 0 | 0 | 0 | 4 |
| Perales et al. (2016) | 1 | 0 | 1 | 0 | 1 | 1 | 0 | 0 | 4 |
| Perrin et al. (2009) | 0 | 0 | 0 | 0 | 1 | 0 | 0 | 0 | 1 |
| Pressler et al. (2013) | 1 | 0 | 0 | 0 | 1 | 1 | 0 | 0 | 3 |
| Pucciarelli et al. (2018) | 1 | 0 | 0 | 1 | 1 | 1 | 1 | 1 | 6 |
| Quinn et al. (2024) | 0 | 0 | 0 | 1 | 1 | 0 | 1 | 1 | 4 |
| Ransmayr et al. (2018) | 1 | 0 | 0 | 0 | 1 | 0 | 0 | 0 | 2 |
| R. Malhotra et al. (2018) | 1 | 0 | 0 | 1 | 1 | 0 | 1 | 1 | 5 |
| Saltz et al. (1999) | 1 | 1 | 1 | 1 | 1 | 0 | 0 | 0 | 5 |
| Siminoff et al. (2024) | 0 | 0 | 0 | 0 | 1 | 0 | 1 | 1 | 3 |
| Snyder & Vitaliano (2020) | 0 | 0 | 1 | 0 | 1 | 0 | 0 | 0 | 2 |
| Van Den Kieboom et al. (2023) | 1 | 0 | 1 | 1 | 1 | 0 | 1 | 1 | 6 |
| Walker et al. (1996) | 0 | 1 | 1 | 1 | 1 | 1 | 1 | 1 | 7 |
| Wen et al. (2022) | 0 | 0 | 1 | 1 | 1 | 0 | 1 | 1 | 5 |

**Supplementary Figure 1: Studies receiving score from each item in NOS**


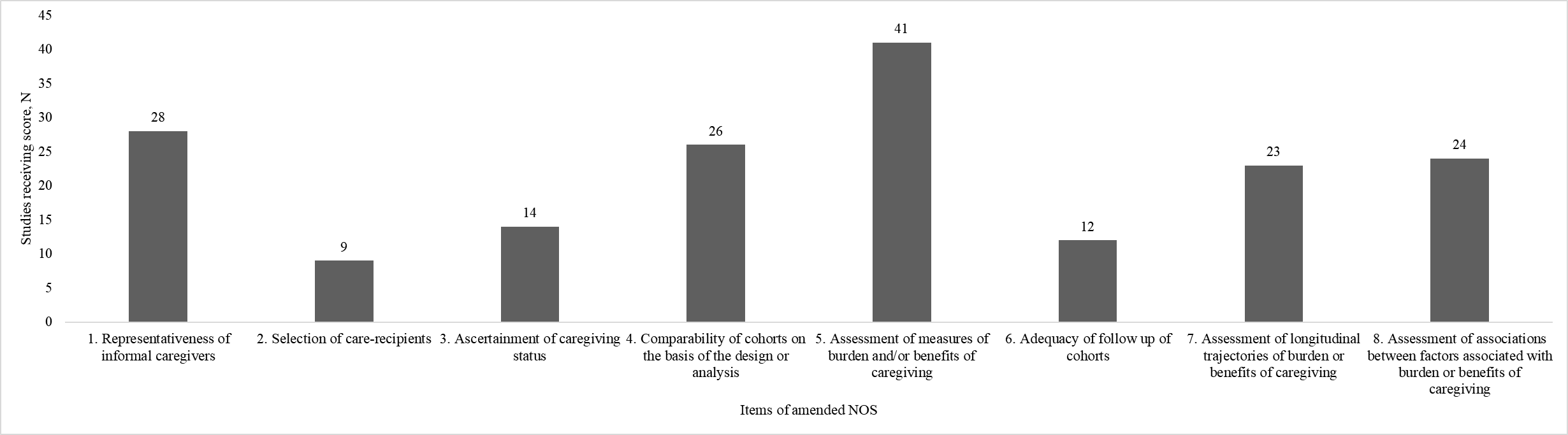


**Supplementary Table 4: Studies on Trajectories of Burden of Caregiving by Care-Recipient Health Conditions**

| **CR diagnosis** | **Trend(s)** | **Study duration** | **Participants following the same average trend, %** | **Measure(s)** | **Score range (Min-Max)** | **Study** |
| --- | --- | --- | --- | --- | --- | --- |
| Dementia | Increase | 15 months | 100.0 | CRA-sd | 5 - 25 | Van Den Kieboom et al. (2023) |
|  |  | 15 months | 100.0 | CRA-lfs | 4 - 20 | Van Den Kieboom et al. (2023) |
|  |  | 15 months | 100.0 | CRA-hp | 5 - 25 | Van Den Kieboom et al. (2023) |
|  |  | 2.0 yrs | 91.7 | RSS | 0 - 60 | Quinn et al. (2024) |
|  |  | 2.0 yrs | 100.0 | ZBI | 0 - 88 | Connors et al. (2023) |
|  |  | 2.0 yrs | 100.0 | ZBI | 0 - 88 | Li et al. (2018) |
|  |  | 2.0 yrs | 100.0 | ZBI | 22 - 110 | Perales et al. (2016) |
|  |  | 3.0 yrs | 100.0 | ZBI | 22 - 110 | Conde-Sala et al. (2014b) |
|  |  | 3.0 yrs | 100.0 | ZBI | 0 - 88 | Connors et al. (2020) |
|  |  | 3.0 yrs | 73.9 | ZBI | 22 - 110 | Conde-Sala et al. (2014a) |
|  | Quadratic Increase | 3.0 yrs | 13.9 | ZBI | 22 - 110 | Conde-Sala et al. (2014a) |
|  | Stable | 1.0 yr | 100.0 | J-ZBI_8 | 0 - 32 | Kajiwara et al. (2018) |
|  |  | 1.0 yr | 100.0 | RC/PROS | 1 - 4 | Bangerter et al. (2019) |
|  |  | 1.0 yr | 100.0 | RC/PROS | 1 - 4 | Liu et al. (2019) |
|  |  | 15 months | 100.0 | CRA-fs | 3 - 15 | Van Den Kieboom et al. (2023) |
|  |  | 2.0 yrs | 100.0 | RS | 0 - 4 | Kuo et al. (2024) |
|  |  | 2.0 yrs | 8.3 | RSS | 0 - 60 | Quinn et al. (2024) |
|  |  | 3.0 yrs | 100.0 | RC/PROS | 1 - 4 | Gaugler et al. (2000) |
|  |  | 5.0 yrs | 100.0 | PROS | 1 - 4 | Mausbach et al. (2007) |
|  | Quadratic Decrease | 3.0 yrs | 12.2 | ZBI | 22 - 110 | Conde-Sala et al. (2014a) |
| Cancer | Increase | 6 months | 100.0 | CRA-lfs | 1 - 5 | Milbury et al. (2013) |
|  | Quadratic Increase | 16 weeks (on average) | 100.0 | CBS-EOLC | 16 - 64 | Guerriere et al. (2016) |
|  | Stable | 6 months | 100.0 | CRA-sd | 1 - 5 | Milbury et al. (2013) |
|  |  | 6 months | 100.0 | CRA-hp | 1 - 5 | Milbury et al. (2013) |
|  |  | 6 months | 90.7 | CRA | 1 - 5 | Lee et al. (2018) |
|  |  | 22 weeks | 100.0 | ZBI | 0 - 88 | Siminoff et al. (2024) |
|  |  | 1.0 yr | 100.0 | CRI-sa | 1 - 4 | Kurtz et al. (2004) |
|  | Decrease | 6 months | 9.3 | CRA | 1 - 5 | Lee et al. (2018) |
|  |  | 6 months | 100.0 | CRA-fs | 1 - 5 | Milbury et al. (2013) |
|  |  | 1.0 yr | 100.0 | CRI-is | 1 - 4 | Kurtz et al. (2004) |
| After surgery | Quadratic Increase | 12 weeks | 100.0 | ZBI/CBI | 0 - 88 | Oakley et al. (2015) |
|  | Stable | 30 days | 100.0 | ZBI | 0 – 88 | Bryson et al. (2013) |
| MCI | Stable | 3.0 yrs | 100.0 | ZBI | 0 - 88 | Connors et al. (2019) |
|  | Increase | 3.0 yrs | 100.0 | ZBI | 0 - 88 | Connors et al. (2023) |
| ADL/IADL limitations | Increase | 2.0 yrs | 100.0 | ZBI | 0 - 88 | Lai (2009) |
| ALS | Stable | 1.0 yr | 100.0 | CBI | 0 - 88 | Goldstein et al. (2006) |
| Heart failure | Decrease | 8 months | 100.0 | OCBS-t/d | 18 - 90 | Pressler et al. (2013) |
| Hip fracture | Decrease | 1.0 yr | 100.0 | CSI | 0 - 13 | Saltz et al. (1999) |
| Stroke | Cubic Decrease | 1.0 yr | 100.0 | CBI | 0 - 100 | Pucciarelli et al. (2018) |

*Note*. CBI = Caregiver Burden Inventory, CBS-EOLC = Caregiver Burden Scale in End-of-Life Care, C-CSI = the Chinese version of Caregiver Strain Index, CR = Care-Recipient, CRA = Caregiver Reaction Assessment, CRA-fs = CRA Financial Strain subscale, CRA-hp = CRA Health Problem subscale, CRA-lfs = CRA Lack of Family Support subscale, CRA-sd = CRA Schedule Disruption subscale, CRI = Caregiver Reaction Inventory, CRI-is = CRI impact on schedule subscale, CRI-sa = CRI sense of abandonment subscale, CSI = Caregiver Strain Index, MCI = Mild Cognitive Impairment, NA = Not Applicable, NR = Not Reported, NS = Not Statistically significant, OCBS-t/d = Oberst Caregiving Burden Scale of perceived difficulty or time with tasks, PROS = the Pearlin's Role Overload Scale, RC = Role Captivity, SCB = Screen of Caregiver Burden, SCQ = Sense of Competence Questionnaire, ZBI = Zarit Burden Interview, J-ZBI_8 = the Japanese version of Zarit Burden Interview, yr(s) = year(s).

**Supplementary Table 5: The Statistics of Trajectories of Burden of Caregiving With Significant Tests for Their Trends in the Included Studies**

| **Study** | **Measure(s)** | **Type of statistics for trajectory(ies)** | | | | | | |
| --- | --- | --- | --- | --- | --- | --- | --- | --- |
| *Studies that delineated average trajectory(ies) by reporting the mean (SD) score at each time point of overall sample* | | *Mean (SD) score at each time point* | | | | | | |
|  |  | *T1* | *T2* | | *T3* | | *T4* | *T5* |
| Bryson et al. (2013) | ZBI (0-88) | 9.3 (9.9) | 8.9 (9.8) | | 8.6 (10.6) | | - | - |
| Conde-Sala et al. (2014b) | ZBI (22-110) | 39.7 (12.4) | 40.3 (11.5) | | 42.7 (13.9) | | 43.5 (13.6) | - |
| Kajiwara et al. (2018) | J-ZBI_8 (0-32) | 11.0 (7.6) | 11.5 (6.4) | | 11.8 (8.2) | | - | - |
| Lai (2009) | ZBI (0-88) | 14.8 (13.9) | 16.2 (15.2) | | 19.7 (19.9) | | - | - |
| Milbury et al. (2013) | CRA-sd (1-5) | 3.0 (0.8) | 2.9 (0.8) | | 2.8 (0.9) | | - | - |
|  | CRA-lfs (1-5) | 1.7 (0.7) | 1.9 (0.7) | | 1.9 (0.8) | | - | - |
|  | CRA-hp (1-5) | 2.0 (0.8) | 2.1 (0.8) | | 2.1 (0.8) | | - | - |
|  | CRA-fs (1-5) | 2.6 (1.0) | 2.3 (1.0) | | 2.3 (1.0) | | - | - |
| Oakley et al. (2015) | ZBI (0-88) | 7.2 (1.0) | 9.1 (1.1) | | 8.6 (NR) | | 8.1 (NR) | - |
|  | CBI (0-96) | 4.7 (0.8) | 8.7 (0.8) | | 6.0 (NR) | | 5.7 (NR) | - |
| Perales et al. (2016) | ZBI (22-110) | 49.5 (NR) | 53.1 (NR) | | 53.8 (NR) | | - | - |
| Pressler et al. (2013) | OCBS-t (18-90) | 40.0 (9.2) | 37.1 (9.1) | | 36.6 (8.1) | | - | - |
|  | OCBS-d (18-90) | 28.9 (8.7) | 27.1 (7.9) | | 26.2 (7.7) | | - | - |
| Pucciarelli et al. (2018) | CBI (0-100) | 25.0 (18.3) | 21.2 (14.0) | | 24.3 (14.8) | | 24.3 (16.3) | 21.9 (13.2) |
| Saltz et al. (1999) | CSI (0-13) | 3.6 (3.7) | 3.0 (3.7) | | 2.9 (3.7) | | - | - |
| Van Den Kieboom et al. (2023) | CRA-sd (5-25) | 15.1 (4.5) | 14.9 (4.3) | | 15.5 (4.6) | | 15.9 (4.8) |  |
|  | CRA-lfs (4-20) | 11.8 (4.2) | 11.8 (4.1) | | 12.4 (3.9) | | 12.4 (4.4) |  |
|  | CRA-hp (5-25) | 10.1 (3.5) | 10.0 (3.4) | | 10.3 (3.3) | | 10.8 (3.7) |  |
|  | CRA-fs (3-15) | 7.2 (2.1) | 7.2 (2.3) | | 7.2 (2.4) | | 7.5 (2.5) |  |
| *Studies that delineated average trajectory(ies) by reporting the baseline mean value(s) and rate(s) of change of overall sample* | | *Baseline value (s), mean (SD)* | | | *Rate (s) of change (SD)* | | | |
| Bangerter et al. (2019) | RC (1-4) | 2.0 (NR) | | | 0.0 (NR) | | | |
|  | PROS (1-4) | 2.8 (NR) | | | 0.0 (NR) | | | |
| Connors et al. (2019) | ZBI (0-88) | 14.7 (14.6) | | | 0.6 (NR) | | | |
| Connors et al. (2020) | ZBI (0-88) | 24.0 (15.8) | | | 0.7 (NR) | | | |
| Gaugler et al. (2000) | RC (1-4) | 2.1 (1.0) | | | 0.0 (NR) | | | |
|  | PROS (1-4) | 2.5 (0.9) | | | 0.0 (NR) | | | |
| Goldstein et al. (2006) | CBI (0-88) | 27.2 (NR) | | | 1.7 (NR) | | | |
| Guerriere et al. (2016) | CBS-EOLC (16-64) | NR | | | linear: 0.02 (NR); quadratic: 0.03 (NR) | | | |
| Kurtz et al. (2004) | CRI-is (1-4) | 2.6 (0.8) | | | -0.1 (NR) | | | |
|  | CRI-sa (1-4) | 1.9 (0.7) | | | 0.0 (NR) | | | |
| Li et al. (2018) | ZBI (0-88) | 21.1 (14.6) | | | 0.8 (NR) | | | |
| Liu et al. (2019) | RC (1-4) | 2.1 (0.8) | | | 0.0 (NR) | | | |
|  | PROS (1-4) | 2.8 (0.6) | | | 0.0 (NR) | | | |
| Mausbach et al. (2007) | PROS (1-4) | NR | | | 0.1 (NR) | | | |
| Siminoff et al. (2024) | ZBI (0-88) | 11.4 (NR) | | | 0.06 (NR) | | | |
| Kuo et al. (2024) | RS (0-4) | 2.1 (NR) | | | -0.06 (NR) | | | |
| Connors et al. (2023) | ZBI (0-88) | 13.8 (12.3) | | | 1.6 (NR) | | | |
|  |  | NR | | | 2.8 (NR) | | | |
| *Studies that reported distinctive trajectories of multiple groups of overall sample* | | *% of participants in each group* | | *Mean score (SD) at each time point* | | | | |
|  |  |  |  | *T1* | | *T2* | *T3* | *T4* |
| Conde-Sala et al. (2014a) | ZBI (22-110) | 73.9 | | 35.9 (NR) | | 36.5 (NR) | 37.5 (NR) | 39.9 (NR) |
|  |  | 13.9 | | 47.1 (NR) | | 60.6 (NR) | 67.7 (NR) | 60.1 (NR) |
|  |  | 12.2 | | 65.7 (NR) | | 52.6 (NR) | 52 (NR) | 51.5 (NR) |
| Lee et al. (2018) | CRA (1-5) | 34.7 | | 2.8 (NR) | | 2.9 (NR) | 2.9 (NR) | 2.9 (NR) |
|  |  | 56.0 | | 2.4 (NR) | | 2.4 (NR) | 2.3 (NR) | 2.4 (NR) |
|  |  | 9.3 | | 1.9 (NR) | | 1.7 (NR) | 1.5 (NR) | 1.6 (NR) |
|  |  |  | | *Intercept* | | *Slope* | | |
| Quinn et al. (2024) | RSS (0-60) | 8.3 | | 36.8 | | -1.0 | | |
|  |  | 46.1 | | 23.6 | | 2.9 | | |
|  |  | 39.5 | | 10.9 | | 2.2 | | |
|  |  | 6.1 | | 10.6 | | 9.9 | | |

*Note*. CBI = Caregiver Burden Inventory, CBS-EOLC = Caregiver Burden Scale in End-of-Life Care, C-CSI = the Chinese version of Caregiver Strain Index, CR = Care-Recipient, CRA = Caregiver Reaction Assessment, CRA-fs = CRA-Financial Strain subscale, CRA-hp = CRA-Health Problem subscale, CRA-lfs = CRA-Lack of Family Support subscale, CRA-sd = CRA-Schedule Disruption subscale, CRI = Caregiver Reaction Inventory, CRI-is = CRI-Impact on Schedule subscale, CRI-sa = CRI-Sense of Abandonment subscale, CSI = Caregiver Strain Index, J-ZBI_8 = the Japanese version of Zarit Burden Interview, NR = Not Reported, OCBS-d = Oberst Caregiving Burden Scale of perceived difficulty with tasks, OCBS-t = Oberst Caregiving Burden Scale of perceived time with tasks, PROS = the Pearlin's Role Overload Scale, RC = Role Captivity, SCB = Screen of Caregiver Burden, SCQ = Sense of Competence Questionnaire, SD = Standard Deviation, ZBI = Zarit Burden Interview.

**Supplementary Table 6: Trajectories of Burden of Caregiving Without Significant Tests for Their Trends in Included Studies**

| **Study** | **Study duration** | **CR diagnosis** | **Measure (s)** | **Score range (Min-Max)** | **Statistical model for trend(s)** | **Trend(s)** | **P-value for trend(s)** | **Type of statistics for trajectory(ies)** | | | | |
| --- | --- | --- | --- | --- | --- | --- | --- | --- | --- | --- | --- | --- |
| *Studies that delineated averaged trajectory(ies) by reporting the mean (SD) score at each time point of overall sample* | | | | | | | | *Mean (SD) score at each time point* | | | | |
|  |  |  |  |  |  |  |  | *T1* | *T2* | *T3* | *T4* | *T5* |
| Alspaugh et al. (1999) | 1.0 yr | Dementia | RC | 1 - 4 | NA | NA | NA | 5.9 (2.5) | 6.0 (2.4) | 6.2 (2.5) | - | - |
|  |  |  | RO | 7 - 28 |  |  |  | 20.6 (3.6) | 20.5 (3.5) | 20.6 (3.4) | - | - |
| Bartoli et al. (2024) | 1.0 yr | Stroke | CBI | 0 - 96 | NA | NA | NA | 24.8 (18.6) | 21.4 (16.5) | 20.7 (17.8) | 28.0 (25.8) | 21.0 (19.5) |
| Brodaty et al. (2014) | 1.0 yr | Dementia | ZBI | 0 - 88 | NA | NA | NA | 22.9 (15.6) | 25.5 (17.2) | 27.7 (19.0) | - | - |
| Burke et al. (2018) | 8 months | ALS | ZBI | 0 - 88 | NA | NA | NA | 26.7 (14.4) | 30.1 (13.6) | 30.6 (13.6) | - | - |
| Han et al. (2017) | 6 months | Stroke | C-CSI | 0 - 13 | NA | NA | NA | 7.4 (2.9) | 7.0 (3.1) | 6.7 (3.1) | 6.3 (3.4) | - |
| Jansen et al. (2021) | 3.0 yrs | Cancer | ZBI-12 | 0 - 48 | NA | NA | NA | 10.3 (7.1) | 10.4 (10.9) | 8.1 (8.1) | - | - |
| Kellermair et al. (2021) | 2.0 yrs | PSP/CBS | ZBI | 0 - 88 | NA | NA | NA | 21.7 (13.6) | 23.6 (16.1) | 26.7 (15.8) | 26.6 (14.9) | 29.2 (17.5) |
|  |  |  | CSI | 0 - 13 | NA | NA | NA | 3.5 (2.8) | 4.4 (3.4) | 4.8 (3.2) | 5.1 (3.2) | 4.7 (2.8) |
| Perrin et al. (2009) | 1.0 yrs | Stroke | SCQ | 27 - 84 | NA | NA | NA | 51.2 (10.8) | 52.1 (11.7) | 51.3 (11.1) | - | - |
| Ransmayr et al. (2018) | 2.0 yrs | Dementia | ZBI | 0 - 88 | NA | NA | NA | 19.3 (14.3) | 20.3 (15.0) | 23.0 (16.2) | 24.4 (16.0) | - |
| Snyder & Vitaliano (2020) | 2.0 yrs | Dementia | SCB | 0 - 75 | NA | NA | NA | 14.1 (10.0) | 16.7 (10.3) | 17.9 (10.4) | - | - |

*Note*. ALS = Amyotrophic lateral sclerosis, CBS = Corticobasal Syndrome, C-CSI = the Chinese version of Caregiver Strain Index, CR = Care-Recipients, CSI = Caregiver Strain Index, NA = Not Applicable, PSP = Progressive Supranuclear Palsy, RC = Role Captivity, RO = Role Overload, SCB = Screen of Caregiver Burden, SCQ = Sense of Competence Questionnaire, SD = Standard Deviation, yr = year, yrs = years, ZBI = Zarit Burden Interview. T1-T4 under the column of “Type of statistics for trajectory(ies)” represented the first to fourth time point of included studies that measured burden of caregiving.

**Supplementary Table 7: Fit Statistics of Group-Based Trajectory Models or Growth Mixture Models Among Studies Identifying Distinctive Trajectories of Burden and/or Benefits of Caregiving**

| **Study** | **Statistical model** | **Group numbers** | **Bayesian information criterion (BIC)** | **Akaike information criterion (AIC)** | **Entropy** | **Average Posterior probability of each group** | **Participants in each group, %** |
| --- | --- | --- | --- | --- | --- | --- | --- |
| *Trajectories of burden of caregiving* | | | | | | |  |
| Conde-Sala et al. (2014a) | GMM | 3 | 7429.1 | 7365 | 0.892 | NR | G1: 73.9; G2: 13.9; G3: 12.2 |
| Lee et al. (2018) | GBTM | 3 | −2007.09 | −1984.51 | NR | NR | G1: 34.7; G2: 56.0; G3: 9.3 |
| Quinn et al. (2024) | GMM | 4 | 18145 | NR | 0.679 | G1: 0.83; G2: 0.83; G3: 0.83; G4: 0.81 | G1: 8.3; G2: 46.1; G3: 39.5; G4: 6.1 |
| *Trajectories of benefits of caregiving* | | | | | | |  |
| R. Malhotra et al. (2018) | GBTM | 2 | −1360.7 | NR | NR | G1: > 0.8; G2: > 0.8 | G1: 41.9; G2: 58.1 |
| Quinn et al. (2024) | GMM | 5 | 17296 | NR | 0.787 | G1: 0.87; G2: 0.90; G3: 0.75; G4: 0.80; G5: 0.79 | G1: 15.2; G2: 67.6; G3: 9.3; G4: 3.4; G5: 4.5 |
| *Joint trajectories of burden and benefits of caregiving* | | |  |  |  |  |  |
| C. Malhotra et al. (2024) | GBMTM | 4 | -3728.1 | NR | NR | NR | G1: 22.7; G2: 28.2; G3: 28.3; G4: 20.8 |
| Quinn et al. (2024) | GMM | 3 | 35389 | NR | 0.891 | G1: 0.96; G2: 0.80; 0.86 | G1: 72.2; G2: 15.2; G3: 12.5 |
| Wen et al. (2022) | GBMTM | 4 | −8145.9 | NR | NR | NR | G1: 38.3; G2: 20.4; G3: 16.4; G4: 24.9 |

*Note*. GBTM = Group-Based Trajectory Model, GMM = Growth Mixture Model, NR = Not Reported.

**Supplementary Table 8: The Statistics of Trajectories of Benefits of Caregiving With Significant Tests for Their Trends in Included Studies**

| **Study** | **Measure** | **Type of statistics for trajectory(ies)** | | | |
| --- | --- | --- | --- | --- | --- |
| *Studies that delineated average trajectory(ies) by reporting the mean (SD) score at each time point of overall sample* | | *Mean (SD) score at each time point* | | | |
|  |  | *T1* | *T2* | *T3* | *T4* |
| Milbury et al. (2013) | CRA-ce (1-5) | 4.3 (0.5) | 4.3 (0.5) | 4.2 (0.5) | - |
| Van Den Kieboom et al. (2023) | CRA-ce (7-35) | 26.3 (4.0) | 26.1 (4.3) | 26.1 (4.3) | 25.8 (4.5) |
| *Studies that delineated average trajectory(ies) by reporting its/their baseline mean value(s) and rate(s) of change of overall sample* | | *Baseline value, mean (SD)* | | *Rate of change (SD)* | |
| Walker et al. (1996) | CS (1-7) | 6.5 (NR) | | -0.2 (NR) | |
| *Study that reported distinctive trajectories of multiple groups of overall sample* | | *% of participants in each group* | *Mean score (SD) at each time point* | | |
|  |  |  | *T1* | *T2* | *T3* |
| R. Malhotra et al. (2018) | PAC (9-54) | 41.9 | 31.2 (NR) | 30.5 (NR) | 29.8 (NR) |
|  |  | 58.1 | 39.5 (NR) | 38.2 (NR) | 36.7 (NR) |
|  |  |  | *Baseline value, mean (SD)* | *Rate of change (SD)* | |
| Quinn et al. (2024) | PAC (9-54) | 15.2 | 39.2 (NR) | -1.5 (NR) | |
|  |  | 67.6 | 28.2 (NR) | 0.2 (NR) | |
|  |  | 9.3 | 15.9 (NR) | 0.6 (NR) | |
|  |  | 3.4 | 14.4 (NR) | 6.8 (NR) | |
|  |  | 4.5 | 28.5 (NR) | -6.3 (NR) | |

*Note.* CGS = Caregiver Gratification Scale, CRI = Caregiver Reaction Inventory, CRA-ce = Caregiver Esteem subscale of Caregiver Reaction Assessment, CS = Caregiving Satisfaction, NR = Not Reported, PAC = Positive Aspects of Caregiving Scale, SD = Standard Deviation.

**Supplementary Table 9: Trajectories of Benefits of Caregiving Without Significant Tests for Their Trends in Included Studies**

| **Study** | **Study**  **duration** | **CR**  **diagnosis** | **Measure** | **Score range**  **(Min - Max)** | **Statistical model for trend(s)** | **Trend(s)** | **P-value for trend(s)** | | **Type of statistics for trajectory(ies)** | | | | | | | |
| --- | --- | --- | --- | --- | --- | --- | --- | --- | --- | --- | --- | --- | --- | --- | --- | --- |
| *Studies that delineated averaged trajectory(ies) by reporting the mean (SD) score at each time point of overall sample* | | | | | | | | *Mean (SD) score at each time point* | | | | | | | |  |
|  |  |  |  |  |  |  |  | *T1* | | *T2* | | *T3* | | *T4* | |  |
| Kajiwara et al. (2018) | 1.0 yr | Dementia | CGS | 0 - 24 | NA | NA | NA | | 14.4 (4.7) | | 14.1 (4.6) | | 13.3 (5.6) | | - | |
| Kurtz et al. (2004) | 1.0 yr | Cancer | CRI-ce | 1 - 5 | NA | NA | NA | | 4.2 (0.6) | | 4.1 (0.6) | | 4.1 (0.6) | | 4.1 (0.6) | |

*Note.* CGS = Caregiver Gratification Scale, CRI-ce = Caregiver Reaction Inventory - caregiver esteem domain, NA = Not Applicable, SD = Standard Deviation, yr = year. T1-T4 represented the first to fourth time point of included studies that measured benefits of caregiving repeatedly.

**Supplementary Table 10: The Statistics of Joint Trajectories of Burden and Benefits of Caregiving**

| **Study** | **Participants in each group, %** | **Measure of burden (Min-Max)** | **Statistics for trajectory(ies) of burden** | **Measure of benefits (Min-Max)** | **Statistics for trajectory(ies) of benefits** |
| --- | --- | --- | --- | --- | --- |
| C. Malhotra et al. (2024) | 22.7% | mCRA (1-5) | Mean values: T1: 2.0, T2: 2.0, T3: 2.0, T4: 2.0, T5: 2.0, T6: 2.0, T7: 2.0. | GAIN (0-40) | Mean values: T1: 35.1, T2: 35.1, T3: 35.3, T4: 35.1, T5: 35.1, T6: 35.2, T7: 35.1. |
|  | 28.2% |  | Mean values: T1: 2.6, T2: 2.6, T3: 2.6, T4: 2.6, T5: 2.6, T6: 2.6, T7: 2.6. |  | Mean values: T1: 28.8, T2: 28.9, T3: 28.9, T4: 28.9, T5: 29.0, T6: 29.0, T7: 29.0. |
|  | 28.3% |  | Mean values: T1: 3.0, T2: 3.0, T3: 3.0, T4: 3.0, T5: 3.0, T6: 3.0, T7: 3.0. |  | Mean values: T1: 36.4, T2: 36.4, T3: 36.3, T4: 36.5, T5: 36.3, T6: 36.4, T7: 36.3. |
|  | 20.8% |  | Mean values: T1: 4.0, T2: 3.9, T3: 3.9, T4: 3.9, T5: 3.9, T6: 4.0, T7: 3.9. |  | Mean values: T1: 31.1, T2: 31.1, T3: 31.0, T4: 31.1, T5: 31.1, T6: 31.1, T7: 31.0. |
| Quinn et al. (2024) | 72.2% | RSS (0-60) | Intercept: 18.8; slope: 4.6 | PAC (9-54) | Intercept: 28.2; slope: 0.6 |
|  | 15.2% |  | Intercept: 15.1; slope: 6.1 |  | Intercept: 39.3; slope: -0.9 |
|  | 12.5% |  | Intercept: 24.2; slope: 4.2 |  | Intercept: 15.5; slope: 3.4 |
| Wen et al. (2022) | 38.3% | mCRA-ish (1-5) | Mean values: T1: 2.5, T2: 2.5, T3: 2.5, T4: 2.5, T5: 2.5, T6: 2.5, T7: 2.5, T8: 2.5, T9: 2.5. | mCRA-ce (1-5) | Mean values: T1: 3.6, T2: 3.6, T3: 3.6, T4: 3.6, T5: 3.6, T6: 3.6, T7: 3.6, T8: 3.6, T9: 3.6. |
|  |  | mCRA-if (1-5) | Mean values: T1: 2.6, T2: 2.6, T3: 2.6, T4: 2.6, T5: 2.6, T6: 2.6, T7: 2.6, T8: 2.6, T9: 2.6. |  |  |
|  |  | mCRA-lfs (1-5) | Mean values: T1: 2.0, T2: 2.0, T3: 2.0, T4: 2.0, T5: 2.0, T6: 2.0, T7: 2.0, T8: 2.0, T9: 2.0. |  |  |
|  | 20.4% | mCRA-ish (1-5) | Mean values: T1: 2.2, T2: 2.2, T3: 2.2, T4: 2.2, T5: 2.2, T6: 2.2, T7: 2.2, T8: 2.2, T9: 2.2. | mCRA-ce (1-5) | Mean values: T1: 4.3, T2: 4.3, T3: 4.3, T4: 4.3, T5: 4.3, T6: 4.3, T7: 4.3, T8: 4.3, T9: 4.3. |
|  |  | mCRA-if (1-5) | Mean values: T1: 2.3, T2: 2.3, T3: 2.3, T4: 2.3, T5: 2.3, T6: 2.3, T7: 2.3, T8: 2.3, T9: 2.3. |  |  |
|  |  | mCRA-lfs (1-5) | Mean values: T1: 1.5, T2: 1.5, T3: 1.5, T4: 1.5, T5: 1.5, T6: 1.5, T7: 1.5, T8: 1.5, T9: 1.5. |  |  |
|  | 16.4% | mCRA-ish (1-5) | Mean values: T1: 3.3, T2: 3.3, T3: 3.3, T4: 3.3, T5: 3.3, T6: 3.3, T7: 3.4, T8: 3.3, T9: 3.3. | mCRA-ce (1-5) | Mean values: T1: 3.3, T2: 3.3, T3: 3.3, T4: 3.3, T5: 3.3, T6: 3.3, T7: 3.3, T8: 3.3, T9: 3.3. |
|  |  | mCRA-if (1-5) | Mean values: T1: 3.5, T2: 3.5, T3: 3.5, T4: 3.5, T5: 3.5, T6: 3.5, T7: 3.5, T8: 3.5, T9: 3.5. |  |  |
|  |  | mCRA-lfs (1-5) | Mean values: T1: 2.7, T2: 2.7, T3: 2.8, T4: 2.8, T5: 2.8, T6: 2.9, T7: 2.9, T8: 2.9, T9: 3.0. |  |  |
|  | 24.9% | mCRA-ish (1-5) | Mean values: T1: 3.4, T2: 3.4, T3: 3.4, T4: 3.4, T5: 3.4, T6: 3.4, T7: 3.4, T8: 3.4, T9: 3.4. | mCRA-ce (1-5) | Mean values: T1: 4.1, T2: 4.1, T3: 4.1, T4: 4.1, T5: 4.1, T6: 4.1, T7: 4.1, T8: 4.1, T9: 4.1. |
|  |  | mCRA-if (1-5) | Mean values: T1: 3.9, T2: 3.9, T3: 3.9, T4: 3.9, T5: 3.9, T6: 3.9, T7: 3.9, T8: 3.9, T9: 3.9. |  |  |
|  |  | mCRA-lfs (1-5) | Mean values: T1: 1.9, T2: 2.0, T3: 2.0, T4: 2.1, T5: 2.1, T6: 2.2, T7: 2.2, T8: 2.3, T9: 2.3. |  |  |

*Note*. GAIN = Gain in Alzheimer Care Instrument, GBMTM = Group-Based Multi-Trajectory Model, GMM = Growth Mixture Model, mCRA = modified Caregiver Reaction Assessment, mCRA-ce = modified Caregiver Reaction Assessment - caregiver esteem subscale, mCRA-if = modified Caregiver Reaction Assessment - impact of finance, mCRA-ish = modified Caregiver Reaction Assessment - impact on schedule and health subscale, mCRA-lfs = modified Caregiver Reaction assessment - lack of family support subscale, NS = Not Statistically significant, PAC = Positive Aspects of Caregiving scale, RSS = Relative Stress Scale. T1-T9 represented the first to ninth time point of included studies that measured burden or benefits of caregiving repeatedly.

**Supplementary Table 11: Caregiver and Care-recipient Characteristics Showing Statistically Significant Associations with the Trajectories of Burden or Benefits of Caregiving in the Included Studies**

| **Study** | **Statistical methods** | **The name of CG and CR characteristics and the interpretation of their associations with the longitudinal trajectories of burden or benefits of caregiving** | **Covariates ^a^** |
| --- | --- | --- | --- |
| ***Burden of caregiving*** | | | |
| Bryson et al. (2013) | GEE | *CG characteristics:* No significant associations reported.  *CR characteristics:* 1) ADL/IADL function (time-invariant): CG caring for CR with worse ADL/IADL function at baseline had a persistently higher burden of caregiving (Coef=0.20, p<0.001). 2) ADL/IADL function (time-varying): CG caring for CR with worse ADL/IADL function between baseline and 30 days postoperative had a faster increase in burden of caregiving from baseline to 30 days postoperative (Coef=0.30, p<0.05). | *CG characteristics*: baseline ADL/IADL function.  *CR characteristics*: None. |
| Conde-Sala et al. (2014a) | MLRM | *CG characteristics (time-invariant or time-varying factors were not specified):* 1) Mental health: CG with better mental health at baseline were less likely to be in the group with the second highest baseline level and quadratically increasing trajectory of burden versus the group with the lowest baseline level and a slightly increasing trend of burden (OR=0.93, p<0.001). 2) Spouse CG: Spouse CG, compared to adult child CG who did not live with CR, were less likely to be in the group with the highest baseline level and quadratically decreasing trend of burden (OR=0.31, p=0.002), and more likely to be in the group with a second highest baseline level and quadratically increasing trend of burden (OR=2.62, p=0.011), versus the group with the lowest baseline level and a slightly increasing trend of burden. 3) Co-residence (Adult child CG): Adult child CG living with CR at baseline, compared to adult child CG who did not live with CR, were more likely to be in the group with the second highest baseline level and quadratically increasing trajectory of burden (OR=6.24, p<0.001), versus the group with the lowest baseline level and a slightly increasing trend of burden. 4) Solo CG: CG who cared for CR alone at baseline, compared to those with additional caregivers, were more likely to be in the group with the second highest baseline level and quadratically increasing trend of burden (OR=3.51, p<0.001), versus the group with the lowest baseline level and a slightly increasing trend of burden.  *CR characteristics (time-invariant or time-varying factors were not specified):* 1) ADL/IADL function: CG caring for CR with better ADL/IADL function at baseline were less likely to be in the group with the highest baseline level and decreasing trend of burden (OR=0.950, p=0.003) or the group with the second highest baseline level and increasing trend of burden (OR=0.960, p=0.001), versus the group with the lowest baseline level and a slightly increasing trend of burden. 2) Neuropsychiatric or behavioral problems: CG caring for CR with more neuropsychiatric or behavioral problems at baseline were more likely to be in the group with the highest baseline level and decreasing trend of burden (OR=1.03, p<0.001), or the group with the second highest baseline level and increasing trend of burden (OR=1.03, p=0.001), versus the group with the lowest baseline level and a slightly increasing trend of burden. | None |
|  |  |  |  |
| Connors et al. (2019) | MEM | *CG characteristics:* 1) Employment (time-invariant): CG who were employed at baseline had a persistently higher burden of caregiving than those who were not employed (Coef=5.80, p=0.011).  *CR characteristics:* 1) Driving status (time-invariant): CG caring for CR who can drive at baseline had a persistently lower burden of caregiving than those of CR who cannot drive (Coef=-4.20, p=0.002). 2) ADL/IADL function (time-invariant): CG caring for a CR with better ADL/IADL function at baseline had a persistently lower burden of caregiving (Coef=-0.20, p=0.001). 3) Neuropsychiatric or behavioral problems (time-invariant): CG caring for a CR with worse neuropsychiatric or behavioral problems at baseline had a persistently higher burden of caregiving (Coef=0.30, p<0.001). | *CG characteristics*: CG-CR relationship, Gender.  *CR characteristics*: Age, Gender, Education, Living status, Number of taken medications. |
| Connors et al. (2020) | MEM | *CG characteristics:* 1) Use of low-level residential care at time diagnosis (time-invariant): CG who used low-level residential care service for CR at time diagnosis had a persistently lower burden of caregiving than CG who did not use any service at home (Coef=-8.90, p=0.003). 2) Male CG (time-invariant): Male CG had a persistently lower burden of caregiving than female CG (Coef=-5.30, p=0.004). 3) Use of home services at time diagnosis (time-invariant): CG who used home-based services at time diagnosis had a persistently higher burden of caregiving than CG who did not use any services at home (Coef=7.90, p<0.001).  *CR characteristics:* 1) Driving status (time-invariant): CG caring for CR who can drive at baseline had a persistently lower burden of caregiving than CG caring for CR who cannot drive (Coef=-2.50, p=0.011). 2) ADL/IADL function (time-invariant): CG caring for CR with better ADL/IADL function at baseline had persistently lower burden of caregiving than those of CR with poorer ADL/IADL function (Coef=-0.20, p<0.001). 3) Number of taken medications (time-invariant): CG caring for CR who took more medications at baseline had a persistently lower burden of caregiving than those of CR who took less (Coef=-0.30, p=0.046). 4) Neuropsychiatric or behavioral problems (time-invariant): CG caring for a CR with worse behavioral and neuropsychiatric problems at baseline had a persistently higher burden of caregiving (Coef=0.30, p<0.001). | *CG characteristics*: CG-CR relationship, CG-CR cohabitation, Employment status.  *CR characteristics*: Age, Gender, Cognitive function, Dementia severity. |
| Connors et al. (2023) | MEM | *CG characteristics:* No significant associations reported.  *CR characteristics:* 1) Apathy (time-invariant): CG caring for CR being more apathy at baseline had persistently higher burden of caregiving (Coef=0.60, p<0.001). | *CG characteristics*: None  *CR characteristics*: Depression, Age, Gender, Antipsychotic, Antidepressant, Number of medications, Incident dementia (at time of MCI diagnosis). |
| Gaugler et al. (2000) | SEM | *CG characteristics:* No significant associations reported.  *CR characteristics:* 1) Neuropsychiatric or behavioral problems (time-varying): CG caring for CR who showed neuropsychiatric or behavioral problems more frequently over time had a faster increase in burden of caregiving than those of CR who showed such problems less frequently (Coef=0.77, p<0.05)*.* | *CG characteristics*: CG-CR relationship.  *CR characteristics*: ADL/IADL function. |
| Guerriere et al. (2016) | MLMC | *CG characteristics:*  1) Male CG (time-invariant): Male CG had persistently lower burden of caregiving than female CG (Coef=-0.26, p<0.001).  2) Education (time-invariant): CG with postgraduate education had persistently higher burden of caregiving than those with education level below postgraduate (Coef=0.19, p<0.05).  3) Caregiving time (time-invariant): CG spending more time in caregiving for CR at baseline had persistently higher burden of caregiving (Coef=0.15, p<0.001).  *CR characteristics:*  1) Male CR (time-invariant): CG caring for a male CR had persistently higher burden of caregiving than those of a female CR (Coef=0.18, p<0.01).  2) Education (time-invariant): CG caring for CR with postgraduate had persistently lower burden of caregiving than those caring for CR with high school or less (Coef=-0.17, p<0.05).  3) Emergency department (ED) visit (time-varying): CG caring for CR with more ED visit had a persistently higher burden of caregiving (Coef=0.05, p<0.001). 4) Days of using hospice care services (time-varying): CG caring for CR who spent more days using hospice care services had a persistently lower burden of caregiving (Coef=-0.04, p<0.05).  5) Physical health (time-varying): CG caring for CR with poorer physical health had a persistently higher burden of caregiving (Coef=0.32, p<0.001). | *CG characteristics*: None.  *CR* *characteristics*: Nursing cost, General practitioner cost, Palliative care physician costs, Days overnight in hospital, Living status (if living alone), Residence location, Comorbidity. |
| Kellermair et al. (2021) | GEE | *CG characteristics:* No significant associations reported.  *CR characteristics:* 1) ADL/IADL function (time-invariant): CG caring for CR with poorer ADL/IADL function at baseline had a persistently higher burden of caregiving (Coef=NR, p<0.001). 2) Neuropsychiatric or behavioral problems (time-invariant): CG caring for CR with more neuropsychiatric or behavioral problems at baseline had a persistently higher burden of caregiving (Coef=NR, p<0.001). | *CG characteristics*: Age, Gender.  *CR characteristics*: Age, Gender, Duration of motor symptoms, Education, Marital status, Frontal Behavioral Inventory (FPI), Frontal Assessment Battery (FAB), Neuropsychiatric Inventory (NPI), Employment status. |
| Van Den Kieboom et al. (2023) | MEM | *CG characteristics:* 1) Competence (time-invariant): CG being more competent at baseline perceived persistently less sense of lacking family support for caregiving (Coef=-0.10, p<0.001) and persistently less physical deterioration due to caregiving (Coef=-0.19, p<0.001). 2) Affective empathy (time-invariant): CG having stronger ability to share the feelings of others at baseline perceived persistently more physical deterioration due to caregiving (Coef=0.05, p=0.006). 3) Age (time-invariant): Older CG at baseline perceived persistently more disruptive schedule due to caregiving (Coef=0.07, p=0.004), and persistently more physical deterioration due to caregiving (Coef=0.04, p=0.033).  4) Spouse CG (time-invariant): Spouse CG perceived persistently more disruptive schedule than adult child or other caregivers (Coef=NR, p=0.006).  5) Education (time-invariant): CG with higher education level at baseline perceived persistently less financial strain in caregiving (Coef=NR, p=0.003). *CR characteristics:* 1) Age (time-invariant): CG caring of an older CR at baseline perceived persistently more financial problems due to caregiving (Coef=-0.04, p=0.003). | None |
| Kuo et al. (2024) | GEE | *CG characteristics:* 1) Predictability (time-invariant): CG who can anticipate their caregiving situations better at baseline had persistently lower burden of caregiving (Coef=-0.08, p<0.001).  *CR characteristics:* 1) ADL/IADL limitations (time-invariant): CG caring for CR with more ADL/IADL limitations at baseline had persistently higher burden of caregiving (Coef=0.01, p<0.001). | *CG characteristics*: Age, Gender, Predictability.  *CR characteristics*: ADL/IADL limitations, Cognitive function. |
| Kurtz et al. (2004) | GEE | *CG characteristics:* No significant associations reported. | *CG characteristics*: Age, Gender, CG-CR relationship, Living arrangement, Education, Caregiver esteem  *CR characteristics*: Age, Gender, Stage of cancer at diagnosis, Comorbidities. |
|  |  | *CR characteristics:* 1) Physical health (time-invariant): CG caring for CR with better physical health at baseline had persistently lower burden of caregiving (Coef=-0.004, p<0.001). 2) Surgery conditions (time-invariant): CG caring for CR who had surgery in the past 39 days before baseline interview had a persistently higher burden of caregiving than those of CR who did not have such surgery (Coef=0.16, p=0.006). 3) Symptom severity related to cancer or treatment (time-invariant): CG caring for CR with more symptoms related to cancer or treatment at baseline had a persistently higher burden of caregiving (Coef=0.01, p=0.021). 4) Depression (time-invariant): CG caring for CR with more depressive symptoms at baseline had a persistently higher burden of caregiving (Coef=0.01, p<0.001). 5) Diagnosis of colon cancer (time-invariant): CG caring for CR with colon cancer at baseline had a persistently higher burden of caregiving than those of CR with breast cancer (Coef=0.35, p=0.004). 6) Diagnosis of lung cancer (time-invariant): CG caring for CR with lung cancer at baseline had a persistently higher burden of caregiving than CG of those with breast cancer (Coef=0.25, p=0.048). |  |
| Lee et al. (2018) | GEE | *CG characteristics (time-invariant or time-varying factors were not specified):* 1) Self-efficacy: CG with a higher self-efficacy at baseline had a persistently lower burden of caregiving (Coef=-0.04, p<0.001). 2) Spouse CG: Spouse CG had a persistently higher burden of caregiving than adult child CG (Coef=0.15, p=0.033). 3) Caring another sick family member: CG caring for an additional sick family member at baseline had a persistently higher burden of caregiving than those who only cared for one CR (Coef=0.19, p=0.001). 4) No alternative CG: CG without an alternative CG at baseline had a persistently higher burden of caregiving than those with an alternative CG (Coef=0.13, p=0.013). 5) Perceived pain: CG who perceived more pain at baseline had a persistently higher burden of caregiving (Coef=0.02, p=0.007). 6) Perceived fatigue: CG who perceived more fatigue at baseline had a persistently higher burden of caregiving (Coef=0.02, p=0.010).  *CR characteristics*: No significant associations reported. | *CG characteristics*: Marital status, Monthly household income.  *CR characteristics*: Gender, Marital status, Employment status, Type of treatment, Physical function, Cancer-related Symptoms severity, Depression. |
| Pucciarelli et al. (2018) | MEM | *CG characteristics:* 1) Male CG (time-invariant): Male CG had a persistently higher burden of caregiving than female CG (Coef=0.24, p=0.026). 2) Co-residence (time-invariant): CG who did not live with CR at baseline had a persistently higher burden of caregiving than those who lived with CR (Coef=0.30, p=0.008).  *CR characteristics*:  1) ADL/IADL function (time-invariant): CG of CR with better ADL/IADL function at baseline had a persistently lower burden of caregiving (Coef=-0.01, p<0.001) | *CG characteristics*, Age, Education level  *CR characteristics*, Age, Gender, Comorbidity. |
| Quinn et al. (2024) | MLRM | *CG characteristics:* 1) Social restriction (time-invariant): CG feeling more difficult to get respite from caregiving at baseline were more likely to be in the group with a persistently higher burden of caregiving (OR=1.55, p<0.05) or being in the group with a faster increase in burden of caregiving over time (OR=1.77, p<0.05). 3) Competence (time-invariant): CG being more competent at baseline were more likely to be in the group with a persistently lower burden of caregiving (OR=0.61, p<0.05). 4) Neuroticism (time-invariant): CG with more neuroticism at baseline (less emotionally stability) were more likely to be in the group with a persistently higher burden of caregiving (OR=1.58, p<0.05). 5) Depression (time-invariant): CG with more depressive symptoms at baseline were more likely to be in the group with a persistently higher burden of caregiving (OR=1.17, p<0.05). 6) CG-CR relationship quality (time-invariant): CG with better relationship with CR at baseline were more likely to be in the group with a persistently lower burden of caregiving (OR=0.79, p<0.05). 7) Self-rated health (time-invariant): CG rating their health better at baseline were more likely to be in the group with a persistently lower burden of caregiving (OR=1.82, p<0.05). 8) Coping (never/sometimes vs often/always, time-invariant): CG perceiving never or sometimes cope well in caregiving at baseline than those feeling often or always coping well in caregiving were more likely to be in the group with persistently higher burden of caregiving (OR=4.11, p<0.05).  9) Self-esteem (time-invariant): CG with better self-esteem at baseline were more likely to be in the group with a persistently lower burden of caregiving (OR=0.79, p<0.05).  10) Male CG (time-invariant): Male CG were more likely to be in the group with a persistently lower burden of caregiving than female CG (OR=2.29, p<0.05).  11) Spouse CG (time-invariant): CG who were relatives or friend of CR were more likely to be in the group with a persistently lower burden of caregiving than spouse CG (OR=2.53, p<0.05).  *CR characteristics:* 1) ADL/IADL function (time-invariant): CG caring for CR with more ADL/IADL limitations at baseline were more likely to be in the group with a persistently higher burden of caregiving (OR=1.09, p<0.05). 2) Neuropsychiatric symptoms (time-invariant): CG caring for CR with more neuropsychiatric symptoms at baseline were more likely to be in the group with a persistently higher burden of caregiving (OR=1.64, p <0.05).  3) Cognitive function (time-invariant): CG caring for CR with better cognitive function at baseline were more likely to be in the group with a persistently higher burden of caregiving (OR=1.09, p<0.05). | *CG characteristics*: Age, Benefits of caregiving.  *CR characteristics*: Types of dementia. |
| Siminoff et al. (2024) | LGCM | *CG characteristics:*  1) Ethnicity (time-invariant): African American CG had persistently lower burden of caregiving than CG with other ethnicity (Coef=-4.40, p<0.001). 2) Spouse CG (time-invariant): Spouse CG had persistently higher burden of caregiving than other CG (Coef=2.00, p<0.05).  *CR characteristics*:  1) Cancer-related symptoms (time-invariant): CG caring for CR with more cancer-related symptoms had persistently higher burden of caregiving (Coef=0.05, p<0.001). | CG characteristics: None |
| ***Benefits of caregiving*** | | | |
| Van Den Kieboom et al. (2023) | MEM | *CG characteristics:* 1) CG-CR relationship quality (time-invariant): CG with better relationship quality with CR at baseline perceived persistently more benefits of caregiving (Coef=0.22, p<0.001). 2) Competence (time-invariant): CG being more competent at baseline perceived persistently more benefits of caregiving (Coef=0.18, p<0.001). | *CG characteristics*: CG-CR relationship quality, Competence, Cognitive and affective empathy, Age, CG-CR relationship, Education, Employment.  *CR characteristics*: Age |
|  |  | *CR characteristics*: No significant associations reported. |  |
| R. Malhotra et al. (2018) | GBTM | *CG characteristics:* No significant associations reported.  *CR characteristics:* 1) ADL/IADL function (time-varying): Among CG who were in the group with persistently high benefits of caregiving, those caring for CR with a poorer ADL/IADL function had persistently higher benefits of caregiving (Coef=0.09, p<0.001); among CG who were in the group with persistently low benefits of caregiving, those caring for CR with a poorer ADL/IADL function had persistently lower benefits of caregiving (Coef=-0.12, p=0.003). | *CG characteristics*: Age, Gender, Ethnicity, Education, Housing types, Chronic disease status, CG-CR relationship, Depressive symptoms, Assistance from a foreign domestic worker.  *CR characteristics*: Age, Depressive symptoms |
| Quinn et al. (2024) | MLRM | *CG characteristics:*  1) Social restriction (time-invariant): CG feeling more difficult to get respite from caregiving at baseline were less likely to be in the group with persistently more benefits of caregiving (OR=0.72, p<0.05). 2) Competence (time-invariant): CG feeling more competence at baseline were more likely to be in the group with persistently more benefits of caregiving (OR=1.71, p<0.05). 3) CG-CR relationship quality (time-invariant): CG with better relationship with CR at baseline were more likely to be in the group with persistently more benefits of caregiving (OR=1.16, p<0.05). 4) Coping (time-invariant): CG perceiving they often or always cope well in caregiving at baseline than those feeling never or sometimes coping well in caregiving were less likely to be in the group with persistently more benefits of caregiving (OR=0.16, p<0.05).  5) Self-esteem (time-invariant): CG with more self-esteem at baseline were more likely to be in the group with persistently more benefits of caregiving (OR=1.10, p<0.05).  6) Depressive symptoms (time-invariant): CG who were more depressed at baseline were more likely to be in the group with persistently less benefits of caregiving (OR=1.95, p<0.05).  *CR characteristics*:  1) Neuropsychiatric symptoms (time-invariant): CG caring for CR with more severe neuropsychiatric symptoms at baseline were more likely to be in the group with persistently less benefits of caregiving (OR=1.08, p<0.05). | *CG characteristics*: Age, Gender, CG-CR relationship, Self-esteem.  *CR characteristics*: Diagnosis of dementia, Cognitive function, ADL/IADL limitations, Neuroticism, Depression, Self-rated health. |
| Walker et al. (1996) | LGCM | *CG characteristics:* 1) Co-residence (time-invariant): CG who lived with CR at baseline had persistently more benefits of caregiving than those who did not (Coef=0.31, p<0.05). | *CG characteristics*: Caregiving duration.  *CR characteristics*: None |
|  |  | *CR characteristics:* 1) ADL/IADL function (time-varying): CG caring for CR with a faster decrease in ADL/IADL function had a faster decline in benefits of caregiving (Coef=-0.64, p<0.01). |  |
| ***Burden and benefits of caregiving*** | |  |  |
| C. Malhotra et al. (2024) | MLRM | *CG characteristics:*  1) CG-CR co-residence (time-invariant): CG living with CR at baseline than those without were more likely to be in the group with persistently higher burden and very high benefits of caregiving (Coef=2.49, p<0.01). 2) Time spent in caregiving (time-invariant): CG spending more caregiving time per day at baseline were more likely to be in the group with persistently higher burden and very high benefits of caregiving (Coef=0.19, p=0.02). 3) Paid for CR treatment with CG’s health saving account (time-invariant): CG using their own health saving account for CR treatment at baseline than those did not were more likely to be in the group with persistently higher burden and lower benefits of caregiving (Coef=1.16, p=0.04). 4) Received emotional support from family (time-invariant): CG having strong emotional support from family at baseline were less likely to be in the group with persistently higher burden and very high benefits of caregiving (Coef=-3.39, P<0.01). 5) Resilience (time-invariant): CG being more resilient at baseline were less likely to be in the group with persistently higher burden and lower benefits of caregiving (Coef=-0.42, p=0.02).  *CR characteristics:*  1) Behavioral problems (time-invariant): CG caring for CR with more behavioral problems at baseline were more likely to be in the group with persistently higher burden and very high benefits of caregiving (Coef=0.12, p<0.01). | *CG characteristics*: Receiving help from a migrant domestic worker, CG-CR relationship, and Ethnicity.  *CR characteristics*: ADL/IADL limitations, Pathological impairment, and Cognitive impairment. |
| Quinn et al. (2024) | MLRM | *CG characteristics:*  1) Competence (time-invariant): CG feeling more competent at baseline were more likely to be in the group with persistently lower burden and more benefits of caregiving (OR=1.65, p<0.05). 2) CG-CR relationship quality (time-invariant): CG having better relationship quality with CR at baseline were more likely to be in the group with persistently lower burden and more benefits of caregiving (OR=1.16, p<0.05). 3) Coping (time-invariant): CG who perceived they never or sometimes cope well in caregiving at baseline than those feeling often or always coping well in caregiving were more likely to be in the group with persistently lower burden and more benefits of caregiving (OR=3.07, p<0.05). 4) Depressive symptoms (time-invariant): CG with more depressive symptoms at baseline were more likely to be in the group with persistently higher burden and less benefits of caregiving (OR=1.03, p<0.05). 5) Social restriction (time-invariant): CG feeling more difficult to get respite from caregiving at baseline were more likely to be in the group with persistently higher burden and less benefits of caregiving (OR=1.40, p<0.05).  6) Male CG (time-invariant): Male CG were more likely to be in the group with persistently less burden and more benefits of caregiving than female CG (OR=1.89, p<0.05).  7) Spouse CG (time-invariant): CG who were relatives or friends of CR were less likely to be in the group with persistently higher burden and less benefits of caregiving than those who were spouses of CR (OR=0.24, p<0.05).  8) Self-esteem (time-invariant): CG with better self-esteem at baseline were more likely to be in the group with persistently lower burden and more benefits of caregiving (OR=1.10, p<0.05).  *CR characteristics:*  1) ADL/IADL function (time-invariant): CG caring for CR with more ADL/IADL impairment were more likely to be in the group with persistently higher burden and less benefits of caregiving (OR=1.02, p<0.05). 2) Neuropsychiatric symptoms (time-invariant): CG caring for CR with more neuropsychiatric symptoms at baseline were more likely to be in the group with persistently higher burden and less benefits of caregiving (OR=1.05, p<0.05). | *CG characteristics*: Age, Gender, CG-CR relationship.  *CR characteristics*: Types of dementia, Neuroticism, Cognitive function, Self-rated health. |
| Wen et al. (2022) | MLRM | *CG characteristics:* 1) Spouse CG (time-invariant): Adult child or other CG than spouse CG were less likely to be in the group with persistently higher burden and high benefits of caregiving (Coef [adult child]=−0.99, p<0.05; Coef [others]=−1.23, p<0.05). 2) Perception of current finances (time-invariant): CG perceiving less financial adequacy at baseline were more likely to be in the group with persistently higher burden and less benefits of caregiving than reference group (Coef[occasionally adequate/don't know]=2.00, p<0.01; Coef[usually inadequate]=1.61, p<0.01).  *CR characteristics*: No significant associations reported. | *CG characteristics*: Age, Gender, Ethnicity, Education, Receiving additional helps, Knowledge of severity of CR’s illness.  *CR characteristics*: None |

*Note*. CG = Caregiver, Coef = Coefficients, CR = Care-Recipient, GBTM = Group-Based Trajectory Model, GEE = Generalized Estimation Equation, LGCM = Latent Growth Curve Model, MEM = Mixed-Effect Model, MLRM = Multinomial Logistic Regression Model, MLMC = Multilevel modelling for change, NA = Not Applicable, OR = Odds Ratio, p = p-value, SEM = Structural Equation Model. Only studies that reported at least one significant association were shown. Effect sizes were limited to two decimal points. In the column of “covariates”, factors were listed if they were included in the multivariable model but did not show statistically significant associations with the trajectory of burden or benefits of caregiving (effect sizes were not reported).

**Section 2: Supplementary Text**

**Newcastle-Ottawa Quality Assessment Scale for Cohort Study (amended)**

**Note**: A study can be awarded a maximum of one star for each item.

**1. Selection**

1) **Representativeness of informal caregivers**

1. Representative of the average informal caregivers in the community *
2. Somewhat representative of the average informal caregivers in the community *
3. Selected group of informal caregivers (e.g., spouse, children, etc)
4. No description of the derivation of the cohort

**Notes**:

- Select A if a study utilized a probability sampling method and recruited two or more types of informal caregivers.
- Select B if a study used a non-probability sampling method and recruited two or more types of informal caregivers.
- Select C if a study exclusively recruited one type of informal caregivers (e.g., only spousal caregivers, or adult child caregivers).
- Select D if a study did not report the sampling method and the composition of informal caregivers.

2) **Selection of the care-recipients**

1. All care-recipients aged 60 or above *****
2. Part of care-recipients aged 60 or above (Mean age > 60)

**Notes**:

- Select A if a study had all care-recipients aged 60 or above.
- Select B if a study reported the mean age of care-recipients larger than 60.

3) **Ascertainment of caregiving status**

1. Provide physical assistance (e.g., support for care-recipients’ ADLs or IADLs) *
2. Provide unpaid care with no description of assistance types *
3. State as informal caregivers while no description of provided care.

**Notes**:

- Select A if a study stated that informal caregivers assisted their care-recipients to perform one or more ADLs or IADLs.
- Select B if a study stated that informal caregivers provided unpaid care or the time of caregiving but did not state their assistance types.
- Select C if a study only stated their participants as informal caregivers with no descriptions of caregiving time and assistance types.

**2. Comparability**

1) **Comparability of cohorts because of the design or analysis**

1. Study included one or more covariates in the statistical analyses *
2. Study only presented unadjusted analyses

**Notes**:

- Select A if a study included one or more covariates in the statistical model for either estimating the trajectories of burden or benefits of caregiving or identifying factors associated with these trajectories.
- Select B if a study only conducted unadjusted analyses.

**3. Outcome**

1) **Assessment of burden and/or benefits of caregiving measures**

1. Independent assessment via validated instruments *
2. Self-report instrument without validation
3. No description

**Notes**:

- Select A if a study used a measurement that reported at least one test of psychometric property (e.g., Cronbach alpha).
- Select B if a study used a measurement but did not report any test of psychometric properties or cited any existing literature.
- Select C if a study did not provide information on burden and/or benefits of caregiving measures.

2) **Adequacy of follow-up of cohorts**

1. Complete follow-up - all subjects accounted for *
2. Incomplete follow-up but the comparison of baseline characteristics between participants with completed data and those without is presented *
3. Incomplete follow-up and the comparison of baseline characteristics between participants with completed data and those without is NOT presented.
4. No description of lost to follow-up.

**Notes**:

- Select A if a study reported findings on a sample with completed data at baseline and all follow-up time points.
- Select B if a study reported findings on a sample with attrition and evaluated the difference in baseline statuses between participants with completed data and those without.
- Select C if a study reported findings on a sample with attrition but did NOT evaluate the difference in baseline statuses between participants with completed data and those without.
- Select D if a study did not report sample attrition and related reasons.

3) **Assessment of longitudinal trajectories of burden or benefits of caregiving:**

1. Used statistical models, such as Generalized Estimating Equations, Mixed-Effect Model, or Latent Growth Curve Model, to estimate a single average trajectory of burden or benefits of caregiving for all study participants combined *
2. Used statistical models, such as Growth Mixed Model, or Group-Based Trajectory Model, to identify multiple groups with distinctive trajectories of burden or benefits of caregiving within the same set of study participants *
3. Reported Mean and Standard Deviation of burden or benefits of caregiving at each time point without applying any statistical model.

**Notes**:

- Select A if a study used the Generalized Estimating Equations, Mixed-Effect Model, or Latent Growth Curve Model and listed a burden or benefits of caregiving measure as the dependent variable.
- Select B if a study used the Growth Mixed Model, or Group-Based Trajectory Model and listed a burden or benefits of caregiving measure as the dependent variable.
- Select C if a study only reported the mean (SD) score of a burden or benefits of caregiving measure at each time point.

4) **Assessment of associations between caregiver and care-recipient characteristics associated with the trajectories of burden or benefits of caregiving:**

1. The statistical test used to analyze the association is clearly described, and the significance and strength of the association are presented, including confidence intervals and/or the probability level (p-value) *
2. The statistical test is not described or incomplete.

**Notes:**

- Select A if a study used the Generalized Estimating Equations, Mixed-Effect Model, or Latent Growth Curve Model, to assess the association between caregiver and care-recipient characteristics and the trajectories of burden or benefits of caregiving. Also, select A if a study used a multinomial logistic regression model to test the association between caregiver and care-recipient characteristics and the groups identified by GMM or GBTM.
- Select B if a study only assessed the association at each time point (e.g., T1 factors associated with T1 burden/benefits, or T1 factors associated with T2 burden/benefits).
